# Gamma Radiation-Induced Oxidation, Doping, and Etching of Two-Dimensional MoS_2_ Crystals

**DOI:** 10.1021/acs.jpcc.0c10095

**Published:** 2021-02-10

**Authors:** Liam H. Isherwood, Gursharanpreet Athwal, Ben F. Spencer, Cinzia Casiraghi, Aliaksandr Baidak

**Affiliations:** † Department of Chemistry, School of Natural Sciences, University of Manchester, Manchester M13 9PL, United Kingdom; ‡ Dalton Cumbrian Facility, Dalton Nuclear Institute, University of Manchester, Cumbria, CA24 3HA, United Kingdom; § Department of Materials, School of Natural Sciences, University of Manchester, Manchester M13 9PL, United Kingdom

## Abstract

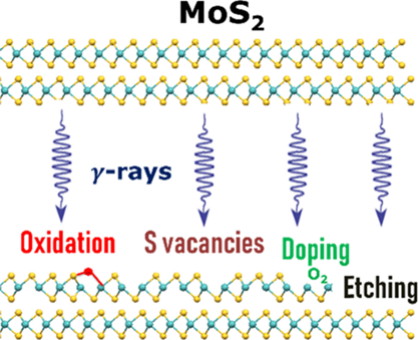

Two-dimensional (2D) MoS_2_ is a promising material for future electronic and optoelectronic applications. 2D MoS_2_ devices have been shown to perform reliably under irradiation conditions relevant for a low Earth orbit. However, a systematic investigation of the stability of 2D MoS_2_ crystals under high-dose gamma irradiation is still missing. In this work, absorbed doses of up to 1000 kGy are administered to 2D MoS_2_. Radiation damage is monitored via optical microscopy and Raman, photoluminescence, and X-ray photoelectron spectroscopy techniques. After irradiation with 500 kGy dose, p-doping of the monolayer MoS_2_ is observed and attributed to the adsorption of O_2_ onto created vacancies. Extensive oxidation of the MoS_2_ crystal is attributed to reactions involving the products of adsorbate radiolysis. Edge-selective radiolytic etching of the uppermost layer in 2D MoS_2_ is attributed to the high reactivity of active edge sites. After irradiation with 1000 kGy, the monolayer MoS_2_ crystals appear to be completely etched. This holistic study reveals the previously unreported effects of high-dose gamma irradiation on the physical and chemical properties of 2D MoS_2_. Consequently, it demonstrates that radiation shielding, adsorbate concentrations, and required device lifetimes must be carefully considered, if devices incorporating 2D MoS_2_ are intended for use in high-dose radiation environments.

## 1 Introduction

Nuclear and space applications are the primary fields that could experience a technological step change due to the implementation of light-weight materials and devices with enhanced capabilities, offered by two-dimensional (2D) materials such as transition metal dichalcogenides (TMDCs). However, the successful deployment of 2D TMDCs in such applications can only be achieved if these materials are resilient and durable upon exposure to high doses of ionizing radiation.^1^ Our work addresses this important question by investigating the gamma-radiation-induced processes in MoS_2_ crystals within the high-dose regime under ambient conditions.

2D TMDCs are a class of layered van der Waals solids with the general formula MX_2_ that exhibit a plethora of magnetic, electronic, and optical properties.^2,3^ Group VI TMDCs, where M = Mo or W and X = S or Se, are semiconductors whose band gaps progressively increase as the crystal thickness is reduced; an indirect–direct transition is observed in monolayer (1 L) crystals.^4,5^ In particular, 1 L MoS_2_ possesses a 1.9 eV direct band gap,^6^ which makes it a promising candidate for photovoltaic applications.^7−9^ With regard to electronic applications, field-effect transistors incorporating 2D MoS_2_ exhibit subthreshold swing values close to the limit of ∼60 mV dec^–1^ at room temperature_,_^10^ while 2D MoS_2_ nanocomposites have proven to be effective electrode materials in Li-ion batteries.^11^ The aforementioned applications are all desirable components of satellite electronics. Moreover, the atomically thin nature and high electron mobility^10^ of 2D MoS_2_ suggest that low-weight devices with minimal power consumption can be fabricated, both of which are prerequisites for satellite instrumentation.

However, in order for a material to be incorporated into applications intended for use in radiation environments, such as the space or nuclear industries, a comprehensive understanding of its radiation damage mechanisms is mandatory. This necessity stems from the adverse effects that the specific radiation field can have on the physical and chemical properties of the material, resulting in the degradation of the device performance. For instance, when a material is irradiated with ions or electrons, atoms can be displaced from their lattice sites due to elastic collisions involving the incident projectiles. Displacement damage must still be considered in the case of gamma irradiation on account of the energetic recoil electrons produced during the Compton scattering of gamma rays. As a result, the vacancies produced by the displacement damage can introduce defect states within the band gap of photoactive materials. These defect states deteriorate the performance of the solar cell by promoting carrier generation, recombination, trapping, and compensation mechanisms.^12^ In addition to displacement damage caused by energetic recoil electrons, the holes produced during the Compton scattering can form oxides and interface charge traps in field-effect transistors. Hence, this ionization damage creates unintended charge concentrations and parasitic fields altering the device performance.^13^

The effect of ion and electron irradiation on the structural,^14−17^ electronic^18−22^ and optical^23−26^ properties of 2D MoS_2_ has been extensively researched.^27^ Only a few studies have evaluated the effect of gamma irradiation on the physical properties of 2D group VI TMDCs. Using a ^60^Co source, Felix et al. irradiated 1 L WS_2_ crystals with an absorbed dose of 400 Gy and observed ferromagnetic hysteresis, which they attributed to a defect configuration involving one W and two S vacancies.^28^ Vogl et al. exposed various 1 L group VI TMDC crystals, produced by micromechanical exfoliation (MME),^29^ to gamma radiation using a ^22^Na source.^30^ Although the photoluminescence (PL) spectrum of MoS_2_, MoSe_2_, and WSe_2_ changed negligibly with increasing dose, a significant linear increase in the PL intensity was observed for WS_2_ crystals as a function of radiation exposure. This was attributed to the passivation of S vacancies by the dissociation of atmospheric O_2_ and its inclusion into the crystal lattice to form WS_2**–***x*_O*_x_* species. Moreover, they fabricated field-effect transistors incorporating 2D MoS_2_ that showed negligible changes in current–voltage characteristics after irradiation with an absorbed dose equivalent to 2170 years at 500 km above the polar caps. Thus, Vogl et al. demonstrated that 2D TMDC devices can successfully withstand the radiation environment of the low Earth orbit.

However, the mean dose rates in the low Earth orbit are typically <20.8 μGy h^–1^,^31^ which are significantly lower than the ∼200 Gy h^–1^ dose rate at the vessel walls of a reactor.^32^ Furthermore, the reprocessing of the spent nuclear fuel and the storage of high-level radioactive waste might see comparably high dose rates. First-principles calculations by Zhang et al. demonstrate that 2D MoS_2_ could be used to sequester problematic Cs, Sr, and Ba radionuclides commonly found in nuclear waste.^33^ In addition to testing the adsorption capacity of MoS_2_, the required experimental studies ought to include an investigation into the radiolytic effects induced by the radioactive decay of the adsorbed nuclides on the surface of MoS_2_ in the presence of water.

Considering an even broader range of applications, a good understanding of the free-radical processes at the MoS_2_–water interface also provides insights into the proposed uses of MoS_2_ as a radioprotector^34^ or, conversely, as a radiosensitizer^35^ for the future clinical treatment of cancer. In addition, ion irradiation has been proven to be an excellent tool to modify 2D materials,^36^ but the full potential of the gamma irradiation technique for controlled defect engineering in TMDCs is yet to be explored.

To the best of our knowledge, just a handful of studies have addressed the effects of the high-dose gamma irradiation of 2D MoS_2_. For example, Ozden et al. utilized a ^60^Co source to irradiate few-layer 2D MoS_2_ films, produced by chemical vapor deposition (CVD),^37^ with an absorbed dose of 1200 kGy under ambient conditions.^38^ They observed the disappearance of the out-of-plane *A*_1g_ and in-plane *E*_2g_^1^ normal vibrational modes and the formation of MoO*_x_* species after irradiation, suggesting a significant increase in the chemical and structural disorder. Similarly, He et al. also utilized a ^60^Co source to irradiate MoS_2_, synthesized via a hydrothermal method, with absorbed doses between 1 kGy and 1000 kGy.^39^ Conversely, they observed (1) no MoO*_x_* formation, (2) an improvement in crystallinity, and (3) red-shifted *A*_1g_ and *E*_2g_^1^ modes with significant intensities after irradiation.

It is evident that a comprehensive, noncontradictory understanding of the effects of the high-dose gamma irradiation on the morphology, vibrational properties, and chemical composition of 2D MoS_2_ is still missing. To address these open questions, we utilized a ^60^Co source to irradiate 2D and bulk MoS_2_ crystals with absorbed doses between 40 and 1000 kGy. Our results show that both S atoms and Mo^IV^ centers at the surface of the MoS_2_ crystals are oxidized upon gamma irradiation under ambient conditions to yield Mo^VI^S*_y_*O*_x_* and sulfate species via a series of intermediates. Regarding the morphology of irradiated MoS_2_, edge-selective radiolytic etching of the uppermost MoS_2_ layers in 2D crystals is observed after irradiation with an absorbed dose of 500 kGy. Concerning radiation-induced changes in the vibrational and optical properties, a blue shift and line width decrease of the *A*_1_^′^ mode in 1 L MoS_2_ is correlated with a blue shift of the PL signal and attributed to p-doping caused by O_2_ adsorption onto vacancies. The observed changes are attributed to the defect production caused by both displacement damage and reactions involving the radiolysis products of adsorbed water and adventitious carbon. Importantly, significant oxidation, etching, and doping of 2D MoS_2_ are observed after an absorbed dose of 500 kGy is administered. This absorbed dose corresponds to ∼104 days at the vessel walls of a reactor;^32^ hence, appropriate consideration must be given to shielding, adsorbate concentrations, and required device lifetimes, if 2D MoS_2_ is intended for use in high-dose radiation environments such as the nuclear industry.

## 2 Methods

### 2.1 Micromechanical Exfoliation

Mono-, bi-, tri-, and quadri-layer and bulk MoS_2_ samples were prepared via the mechanical cleavage of molybdenite crystals (Manchester Nanomaterials) using dicing tape. The exfoliated flakes were deposited onto SiO_2_/Si substrates (IDB Technologies, oxide thickness ca. 300 nm), which had been sonicated in acetone (10 min) and propan-2-ol (5 min) before being dried under a nitrogen flow. Prior to deposition, the substrates were heated to 130 °C under ambient conditions to suppress the formation of an interfacial water layer. Optical microscopy was used to identify monolayer and few-layer flakes on account of their distinctive optical contrast. The thicknesses of the MoS_2_ flakes were then confirmed via Raman spectroscopy.

### 2.2 Chemical Vapor Deposition

A 1 cm × 1 cm polycrystalline film of the monolayer MoS_2_ on SiO_2_/Si (2DLayer, Atomix Inc., Durham, NC) was cut into four 5 mm × 5 mm sections using a glass scribe. Prior to irradiation, each sample was characterized via Raman and photoluminescence spectroscopy techniques.

### 2.3 Gamma Irradiation

Gamma irradiations were performed using a self-contained Foss Therapy Service Inc. 812 ^60^Co source located at the Dalton Cumbrian Facility, University of Manchester. The dose rates administered to the samples varied between 265 and 365 Gy/min, determined using the ionization chamber detector. Prior to irradiation, the MoS_2_ samples were transferred into crimp-sealed borosilicate vials (10 mL capacity) under ambient conditions. The absorbed doses administered to the MoS_2_ samples prepared via micromechanical exfoliation and chemical vapor deposition varied from 40 kGy up to 1000 kGy. The sample used to investigate the influence of adsorbed water on the radiation damage mechanisms in MoS_2_ was placed inside a constant humidity chamber that contained a beaker with pure water. This environment possessed a relative humidity of about 95%. The sample was conditioned for 5 days inside the chamber prior to irradiation. The irradiation took place immediately after the sample was taken from the constant humidity chamber.

### 2.4 Raman Spectroscopy

Raman measurements were acquired in backscattering geometry under ambient conditions using a WITec Alpha300 spectrometer equipped with 1800 lines mm^–1^ grating and a 100× objective lens (numerical aperture = 0.95), resulting in a spectral resolution of ∼1 cm^–1^ and a spatial resolution of ∼330 nm. An excitation wavelength of 514.5 nm was used for all measurements, and laser power was kept below 0.15 mW to avoid thermal damage or local heating effects. WITec Project 2.08 was used to fit all Raman signals using Lorentzian line shapes and to create Raman maps.

### 2.5 Photoluminescence Spectroscopy

PL measurements were also acquired under ambient conditions using a WITec Alpha300 spectrometer equipped with a 514.5 nm laser, operated below 0.15 mW, and a 100× objective lens. However, 600 lines mm^–1^ grating was used to increase the spectral window, enabling the measurement of both the A^–^ trion and the B exciton at higher energies. Data processing is analogous to that described for Raman measurements with the exception that Gaussian line shapes were used on account of the broader PL signals.

### 2.6 X-ray Photoelectron Spectroscopy

An Axis Ultra Hybrid (Kratos Analytical) equipped with an Al Kα X-ray source using 10 mA emission and operating at 15 kV bias was used to obtain photoelectron spectra. Pass energies of 20 and 80 eV were used for high-resolution spectra and survey scans, respectively. The typical vacuum level for measurements was between 2 × 10^–8^ and 3 × 10^–8^ mbar. The spectra were analyzed using CasaXPS software and are calibrated to the C 1s signal of adventitious carbon at 284.8 eV.

## 3 Results and Discussion

We investigated two types of samples: MoS_2_ flakes produced by MME, which contain a mixture of mono- (1 L), bi- (2 L), tri- (3 L), quadri-layer (4 L), and bulk single crystals, as identified by Raman spectroscopy;^40^ and commercially available polycrystalline 1 L films, produced by CVD. In both cases, MoS_2_ is deposited onto SiO_2_/Si wafers. The thickness of 1 L, 2 L, and 3 L MoS_2_ crystals can be determined unambiguously by calculating the frequency difference between the *A*_1g_ and *E*_2g_^1^ modes using a well-established procedure based on Raman spectroscopy.^40^ Bulk crystals are easily identified by their optical contrast, while 4 L thicknesses are assigned to crystals exhibiting an *A*_1g_ and *E*_2g_^1^ frequency difference of 23.8 cm^–1^, in accordance with previous correlative atomic force microscopy measurements.^41^

For irradiation, MoS_2_ samples were placed into borosilicate vials and sealed under ambient conditions. Gamma radiation was administered homogeneously throughout the MoS_2_ samples using gamma photons from a ^60^Co source. X-ray photoelectron spectroscopy (XPS) was then used to evaluate the radiolytic oxidation of MoS_2_, while correlative optical microscopy and Raman and PL spectroscopy maps were utilized to gain insights into the radiation-induced changes in the morphology as well as the vibrational and optical properties of MoS_2_.

### 3.1 Radiolytic Oxidation of MoS_2_

**Figure 1:**
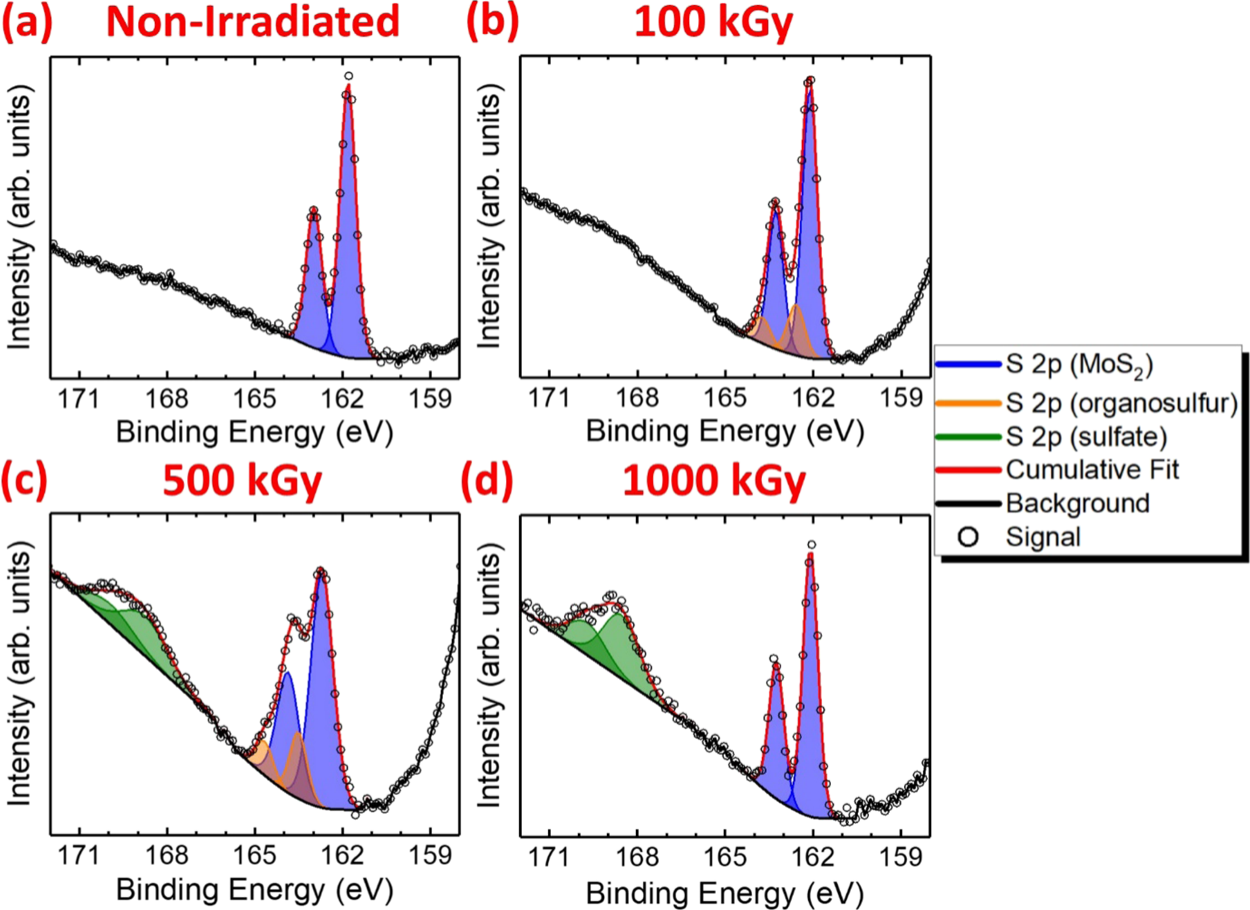
Deconvoluted S 2p X-ray photoelectron spectra of MoS_2_ crystals, deposited by micromechanical exfoliation: (a) prior to irradiation and after irradiation with the absorbed doses of (b) 100 kGy, (c) 500 kGy, and (d) 1000 kGy.

Figure 1 shows the deconvoluted S 2p XPS spectra of pristine and irradiated MoS_2_ crystals, prepared via MME, up to an absorbed dose of 1000 kGy. The S 2p spectrum of nonirradiated MoS_2_ consists of a single doublet, in which the S 2p_3/2_ and S 2p_1/2_ photoelectron lines exhibit binding energies of 161.8 and 163.0 eV, respectively, corresponding to the S^2–^ sulfide environment of MoS_2_ (Figure 1a). After irradiation with an absorbed dose of 100 kGy, a second doublet is observed at a higher binding energy in which the S 2p_3/2_ and S 2p_1/2_ photoelectron lines exhibit values of 162.6 and 163.8 eV (Figure 1b). This doublet is attributed to the formation of organosulfur species, which could contain, for example, S–O, S–C, or S–H covalent bonds. Photoelectrons originating from this new S environment exhibit lower kinetic energies, i.e., possess higher binding energies, on account of the higher electronegativity of O, C, and H atoms relative to Mo^IV^ centres.^42^ This results in the organosulfur S 2p core level electrons experiencing a greater effective nuclear charge due to the reduction in the S atom electron density.

Concerning the possible mechanism of organosulfur formation, such compounds could be the stable products of reactions involving S atoms at the MoS_2_ crystal surface and the radiolysis products originating from adsorbed water or adventitious carbon, both of which are ubiquitous adsorbates on air-exposed surfaces under ambient conditions.^43−45^ For instance, water radiolysis generates a mixture of highly reactive radical and molecular species, such as the hydrated electron (e^–^_aq_), the hydroxyl radical (OH), and hydrogen peroxide (H_2_O_2_).^46^ These species are capable of initiating a plethora of chemical reactions directly involving or affecting MoS_2_. Moreover, the ionization and excitation of adsorbed hydrocarbons yield reactive carbon-centered radicals.^47^ Subsequent reactions of these species with the MoS_2_ crystal surface could easily lead to the formation of the observed organosulfur compounds.

Regarding the chemical nature of the organosulfur compounds, the presence of sulfoxide moieties can be ruled out as the binding energy of the S 2p_3/2_ photoelectron line in sulfoxide compounds is ∼165.8 eV,^48,49^ which is ∼3.2 eV higher than the organosulfur S 2p_3/2_ signal observed in MoS_2_ after 100 kGy gamma irradiation (Figure 1b). However, the S 2p_3/2_ photoelectron lines of thiol and aliphatic sulfide species exhibit binding energies between 163 and 164 eV.^49,50^ This energy range is in reasonable agreement with the value of 162.6 eV observed for the organosulfur species in Figure 1b, when taking into account the electropositive influence of the Mo^IV^ center(s) still bonded to the S atom. According to the literature, defective 1 L MoS_2_ crystals with S vacancies at the surface are indeed capable of reacting with organic (1-butanethiol) molecules to produce stable crystals containing Mo^IV^–S–C_4_H_9_ moieties, i.e., the crystal surface is functionalized with alkyl chains.^51^

Interestingly, after administering an absorbed dose of 500 kGy, the binding energy of the organosulfur S 2p core level electrons increases by 0.9 eV, such that the S 2p_3/2_ and S 2p_1/2_ signals exhibit binding energies of 163.5 and 164.7 eV, respectively (Figure 1c). Due to this binding energy increase, the organosulfur photoelectron lines now agree well with the values reported for aliphatic sulfides and thiols,^49,50^ suggesting further Mo^IV^–S bond scission at higher absorbed doses, i.e., some S atoms possibly becoming fully detached from the crystal lattice. Moreover, a new doublet is observed in which the S 2p_3/2_ and S 2p_1/2_ photoelectron lines exhibit binding energies of 168.7 and 169.9 eV, respectively. These values agree well with those reported for sulfate-containing compounds.^52^ It is reasonable to suggest that the products of water radiolysis, in particular, the strongly oxidizing hydroxyl radical (·OH) and hydrogen peroxide (H_2_O_2_), could facilitate the oxidation of S atoms to produce SO_4_^2–^ species.

In addition to the oxidizing species such as ·OH and H_2_O_2_, strongly reducing hydrated electrons, e^–^_aq_, are also generated during water radiolysis; the radiation chemical yields of ·OH and e^–^_aq_ are equal in deaerated water.^46^ In our experiments, one can expect the water layers to be adsorbed on the surface of the MoS_2_ crystals due to the finite humidity of the atmosphere. Naturally, in the presence of air, the adsorbed water will also contain dissolved oxygen. It is well known that O_2_ acts as an efficient scavenger for e^–^_aq_, yielding the superoxide radical anion via O_2_ + e^–^_aq_ →·O_2_^–^ reaction. Although ·O_2_^–^ generally acts as a reducing agent,^53^ its reducing ability is significantly smaller than that of the parent e^–^_aq_.^54,55^ The atomic hydrogen, H, which is another primary reducing species produced during water radiolysis,^54^ is also readily scavenged by O_2_ to produce the hydroperoxyl radical via O_2_ +·H →·O_2_H reaction. Due to its p*K*_a_ value of 4.8, in neutral aqueous media, ·O_2_H will exist mostly as ·O_2_^–^ through the equilibrium:·O_2_H + OH^–^ ⇌ O_2_^–^ + H_2_O.

In essence, primary strongly reducing species from water radiolysis become rapidly scavenged by O_2_ to yield weakly reducing·O_2_^–^ species, creating overall oxidizing conditions (note that the·OH and H_2_O_2_ remain available for reacting with MoS_2_), thus promoting the oxidation of sulfur atoms in MoS_2_. This process is expected to proceed through a sequence of electron donations since the oxidation state of the sulfur atom needs to change, eventually, from −2 to +6. For such extensive oxidation to occur, large absorbed doses of radiation would be required. Indeed, in our experiments, the formation of sulfate species becomes observable only after irradiation with an absorbed dose of 500 kGy and is attributed to the cumulative effect of oxidation reactions between OH, H_2_O_2_, and the S atoms as well as the aliphatic sulfides/thiols produced at the earlier stages of radiolysis. In good accordance with our studies, Lefticariu et al. unambiguously observed sulfate formation in aqueous systems containing FeS_2_, which they attributed to oxidation reactions involving the products of water radiolysis.^56^

After an absorbed dose of 1000 kGy is administered to the MoS_2_ crystals, the organosulfur S 2p doublet is no longer observed (Figure 1d). This is accompanied by a significant increase in the integrated intensity of the sulfate doublet relative to the S^2–^ environment. This is expected since the C–S bond cleavage and photooxygenation of aliphatic sulfides/thiols^57^ are mediated by the products of water radiolysis to result in sulfate species being a prevailing oxidation product at higher absorbed doses. Therefore, sulfate species represent the final product of S atom oxidation, with the organosulfur compounds being intermediates. The sampling depth of the Al Kα X-ray source utilized for XPS measurements is ∼6 nm,^58,59^ corresponding to the uppermost ∼8 MoS_2_ monolayers without accounting for adsorbates. Therefore, the absence of organosulfur intermediates and the increased concentration of sulfate species suggest extensive oxidation of the MoS_2_ crystal surface upon irradiation with a large absorbed dose of 1000 kGy.

**Figure 2:**
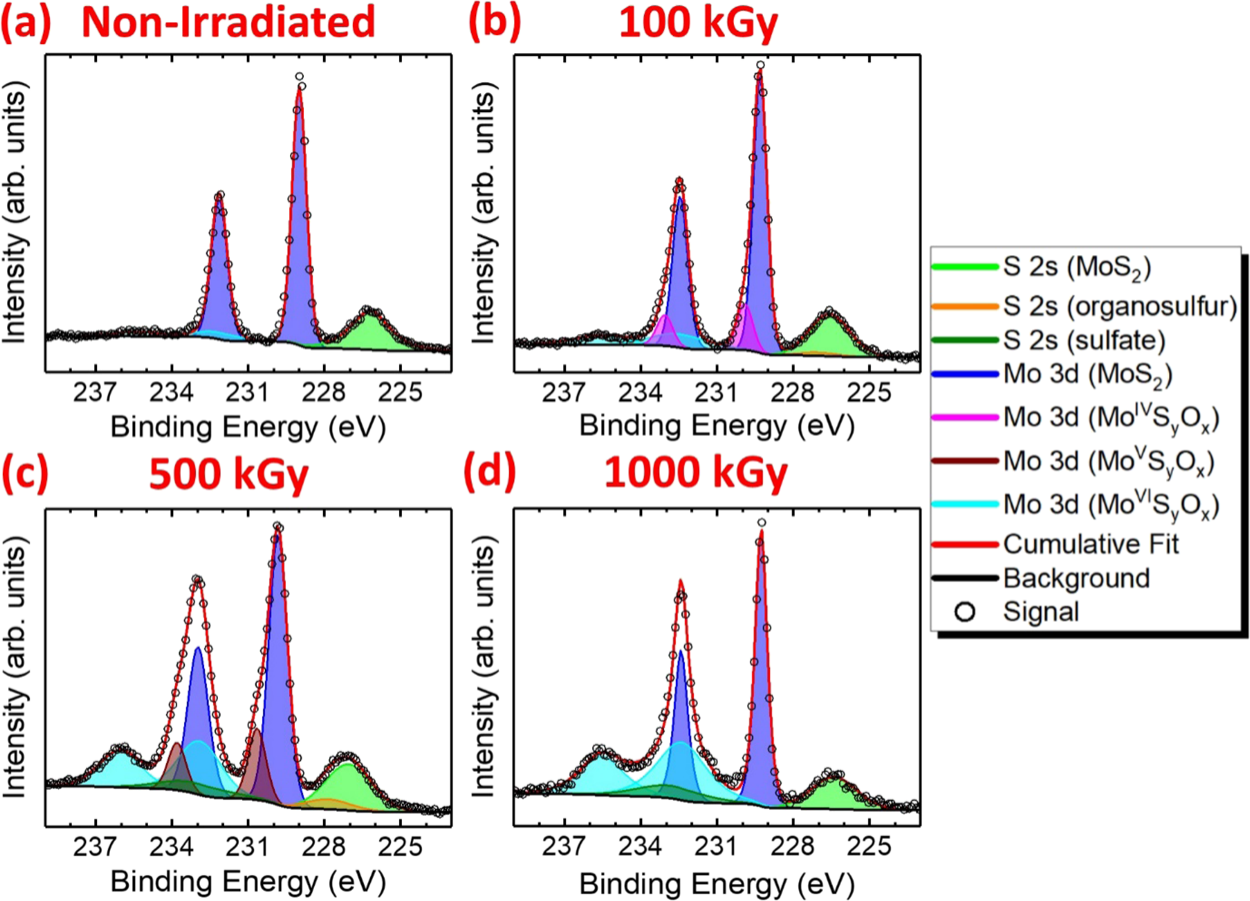
Deconvoluted Mo 3d X-ray photoelectron spectra of MoS_2_ crystals, deposited by micromechanical exfoliation: (a) prior to irradiation and after irradiation with absorbed doses of (b) 100 kGy, (c) 500 kGy, and (d) 1000 kGy.

Figure 2 shows the deconvoluted Mo 3d XPS spectra of pristine and irradiated MoS_2_ crystals, prepared via MME, up to an absorbed dose of 1000 kGy. The Mo 3d spectrum of nonirradiated crystals consists of one singlet and one doublet in which the S 2 s, Mo 3d_5/2_, and Mo 3d_3/2_ photoelectron lines, corresponding to the S^2–^ and Mo^IV^ environments of MoS_2_, exhibit binding energies of 226.2, 229.0, and 232.1 eV, respectively (Figure 2a). After irradiation with an absorbed dose of 100 kGy, the formation of a second doublet is observed at a higher binding energy in which the Mo 3d_5/2_ and Mo 3d_3/2_ photoelectron lines exhibit values of 229.9 and 233.1 eV (Figure 2b). This doublet is attributed to the formation of Mo^IV^S*_y_*O*_x_* species, i.e., formation of Mo–O bonds where O^2–^ replaces S^2–^, but the oxidation state of the Mo center remains +4. The binding energy of the Mo 3d_5/2_ photoelectron signal is in good agreement with that reported for MoO_2_;^60^ however, as the exact stoichiometry of the oxidized Mo^IV^ product is hard to deduce, the general formula Mo^IV^S*_y_*O*_x_* is assigned to this species in our work.

The mechanism of the Mo–O bond formation is likely to proceed via the displacement of S atoms from their lattice sites due to elastic collisions involving energetic recoil electrons from the Compton scattering of gamma photons. Calculated recoil electron energies, as a function of the scattering angle, suggest that the production of S vacancies is energetically feasible when using a ^60^Co source (Figure S1a, Supporting Information). Furthermore, the normalized differential cross-section dictates that ∼68% of all recoil electrons produced during the Compton scattering will have sufficient kinetic energy to displace S atoms from their lattice sites (Figure S1b, Supporting Information).

Density functional theory calculations show that when atmospheric O_2_ molecules are adsorbed onto S vacancies, the activation energy barrier for O_2_ dissociation is lowered, which could facilitate Mo–O bond formation.^61^ In addition to spontaneous O_2_ dissociation, the ionization of adsorbed O_2_ due to the Compton scattering will produce highly reactive ·O_2_^+^ species.^62^ Moreover, reactions between Mo^IV^ centers, bearing vacant coordination sites, and ·OH and H_2_O_2_ species, formed during water radiolysis, may also contribute toward Mo–O bond formation. Indeed, passivation of S vacancies in defective MoS_2_ via inclusion of O atoms from H_2_O_2_ has been observed experimentally.^63^

After irradiation with an absorbed dose of 500 kGy, the binding energy of the Mo^IV^S*_y_*O*_x_* doublet further increases by ∼0.7 eV such that the Mo 3d_5/2_ and Mo 3d_3/2_ photoelectron lines exhibit binding energies of 230.7 and 233.8 eV, respectively (Figure 2c). The magnitude of this binding energy increase suggests that Mo^IV^ centers undergo further oxidation to yield Mo^V^S*_y_*O*_x_* species. Indeed, the binding energies observed in this work agree well with those observed for Mo^V^ centers in oxysulfide thin films.^64^ Moreover, the integrated intensity of a third Mo 3d doublet becomes considerable after irradiation with an absorbed dose of 500 kGy. The doublet exhibiting Mo 3d_5/2_ and Mo 3d_3/2_ binding energies of 232.9 and 236.1 eV, respectively, is attributed to the formation of Mo^VI^S*_y_*O*_x_* species. These binding energies match well with those reported for MoO_3_.^65,66^ Since the exact stoichiometry of the oxidized Mo^VI^ species is difficult to deduce, the general formula Mo^VI^S*_y_*O*_x_* is used to denote this oxidized product. In addition to the formation of the oxidized Mo^V^ and Mo^VI^ species, two singlet photoelectron lines are observed at 227.9 and 233.1 eV, corresponding to the binding energies of the organosulfur and sulfate S 2s core level electrons, respectively.

Akin to the absence of the organosulfur photoelectron lines in the S 2p XPS spectrum of MoS_2_ irradiated with an absorbed dose of 1000 kGy, no Mo^V^S*_y_*O*_x_* species are observed in the corresponding Mo 3d spectrum (Figure 2d). Equivalent to sulfate formation, the oxidation of Mo atoms to Mo^VI^S*_y_*O*_x_* and MoO_3_ represents the final product of MoS_2_ oxidation.

Similar to how organosulfur intermediates appear to be formed via reactions involving the products of adventitious carbon radiolysis, it is possible that Mo^V^S*_y_*O*_x_* intermediates may be the product of reactions between S vacancies and either water radiolysis products and/or oxygen. This hypothesis was tested by irradiating MoS_2_ crystals, fabricated by MME, with a moderate absorbed dose of 100 kGy, after the sample had been conditioned in a constant humidity chamber for 5 days at 95% relative humidity prior to irradiation. The deconvoluted XPS spectra (Figures S2 and S3, Supporting Information) indeed show strongly enhanced formation of Mo^V^S*_y_*O*_x_* in this sample proving that the Mo^V^S*_y_*O*_x_* species is an important oxidation intermediate produced via the reaction of MoS_2_ surface atoms with the oxidizing products from water radiolysis. This result also highlights an important role which water plays in the acceleration of the radiolytic oxidation of 2D MoS_2_.

Finally, to assess the effect of crystal morphology on the radiolytic processes in MoS_2_, polycrystalline 1 L CVD films of MoS_2_ were irradiated with absorbed doses between 40 kGy and 150 kGy. Their XPS spectra are shown in Figures S4 and S5, Supporting Information. In these samples, extensive oxidation of MoS_2_, resulting in Mo^VI^S*_y_*O*_x_* and sulfate formation, is observed even within the low-dose regime, contrasting the results obtained for MME samples, which showed much milder oxidation under the same doses. This difference is attributed to the polycrystalline morphology of the 1 L CVD films, i.e., they contain a greater number of active edge sites and grain boundaries relative to their predominantly single-crystal MME counterparts. An important role of the active edge sites in the radiolytic transformation of MoS_2_ crystals is discussed in detail next.

### 3.2 Radiation Effects on MoS_2_ Morphology

**Figure 3:**
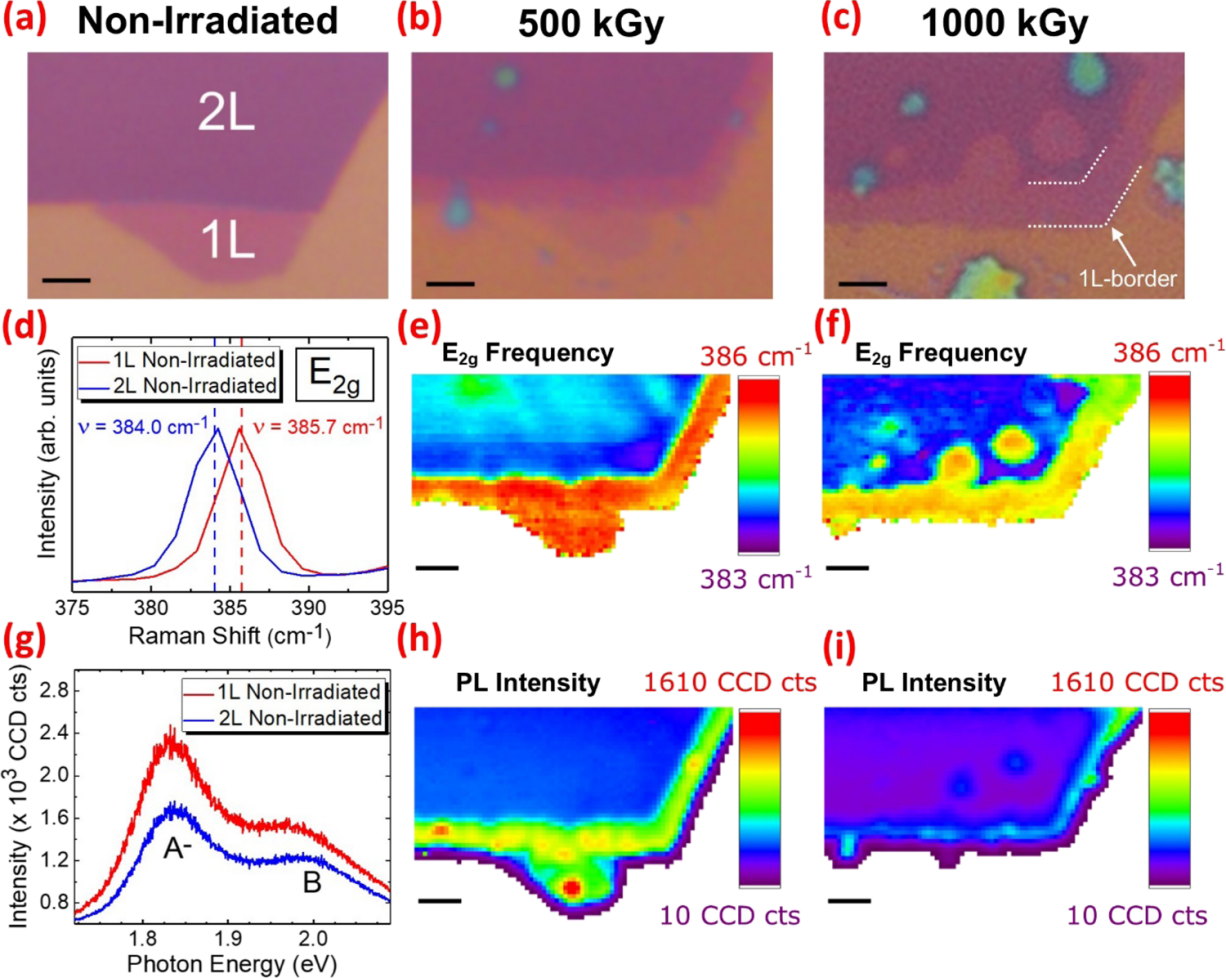
Top row: Optical micrographs of monolayer (1 L) and bilayer (2 L) crystals, produced by micromechanical exfoliation, (a) prior to irradiation and after irradiation with absorbed doses of (b) 500 kGy and (c) 1000 kGy. Middle row: (d) Raman spectra of nonirradiated MoS_2_ crystals showing a blue shift of the in-plane *E*_2*g*_^1^ mode when the thickness decreases from 2 L (blue) to 1 L (red) and correlative Raman maps of the 1 L and 2 L domains visible in the optical micrographs showing the variation of the *E*_2g_^1^ frequency across the crystals after irradiation with absorbed doses of (e) 500 kGy and (f) 1000 kGy. Bottom row: (g) photoluminescence (PL) spectra of nonirradiated MoS_2_ crystals showing an increase in the A^–^ trion and B exciton intensities when the thickness decreases from 2 L (blue) to 1 L (red) and correlative PL maps of the 1 L and 2 L domains visible in the optical micrographs showing the variation of the PL intensity across the crystals after irradiation with absorbed doses of (h) 500 kGy and (i) 1000 kGy. All scale bars correspond to a length of 2 μm.

Concerning the effects of high-dose gamma irradiation on the morphology of 2D MoS_2_ produced by MME, Figure 3a shows an optical micrograph of 1 L and 2 L crystals prior to irradiation. Differentiation between the SiO_2_ substrate and the 1 L/2 L MoS_2_ regions is facile on account of the distinct optical contrast and sharp, well-defined edges of the crystals. After irradiation with an absorbed dose of 500 kGy, the size of the 1 L crystal domain is significantly reduced (Figure 3b).

Moreover, the optical contrast of the 2 L domain is considerably lower at the periphery of the crystal, such that it resembles the contrast of the 1 L crystal prior to irradiation. In other words, after irradiation, the surface of the 2 L crystal appears to be “etched”, yielding 1 L MoS_2_. These findings suggest that the edges of the uppermost layers in MoS_2_ crystals are more susceptible to radiation damage, resulting in observed reduction in the lateral dimensions of 1 L crystals and the formation of a “1 L border” in 2 L crystals.

The higher susceptibility of edge and surface atoms toward radiation damage could be attributed to the high reactivity of edge defects as well as the spatial confinement of adsorbates. More precisely, the concentration of vacant coordination sites, localized at the edges of MoS_2_ crystals, has been correlated with the catalytic efficiency of MoS_2_ in the hydrogen evolution reaction.^67,68^ If such active edge sites exhibit higher reactivity than their basal plane counterparts, it follows that the rate of reactions involving the products of adsorbate radiolysis could also be significantly higher at the edges of MoS_2_ crystals.

1 L MoS_2_ crystals are just 0.7 nm thick. Hence, they are visible via optical microscopy only when deposited on substrates with discrete dielectric thicknesses. In this work, MoS_2_ crystals are deposited on top of 300 nm thick quartz-coated silicon wafers. Consequently, the optical contrast of deposited 2D MoS_2_ crystals is significantly increased due to multiple reflections at various interfaces,^69^ and 1 L crystals become easily visible (Figure 3a). Upon exposure to gamma irradiation, the active edge sites of 1 L MoS_2_ crystals react with the products of adsorbate radiolysis, yielding oxidized intermediates and, eventually, Mo^VI^S*_y_*O*_x_* and sulfate species, as discussed earlier. In contrast, these oxidized products are not visible via optical microscopy as they are formed in the amorphous state. As the crystalline interface becomes more disordered, fewer reflections are possible, leading to a significant reduction in optical contrast. Hence, the 1 L crystal domain size appears to “shrink” (Figure 3b). Moreover, the spatial distribution of adsorbates is such that they are confined to the surface, i.e., the uppermost layer of the MoS_2_ crystal. Therefore, the rate of reaction between the products of adsorbate radiolysis and the bottom layer of the 2 L MoS_2_ crystal would be considerably lower due to the barrier of amorphous products formed during the oxidation of the top layer of the 2 L crystal. A combination of these processes is likely to be responsible for the appearance of a 1 L-border after irradiation with an absorbed dose of 500 kGy (Figure 3b).

Furthermore, in the optical micrograph of the same 1 L and 2 L crystals after irradiation with an absorbed dose of 1000 kGy, three circular regions appear in the 2 L crystal domain, exhibiting significantly reduced optical contrast (Figure 3c). This observation suggests that holes have been etched in the uppermost MoS_2_ layer of the 2 L crystal. The formation of these circular features indicates a transition to nonselective radiolytic etching in MoS_2_ crystals irradiated with absorbed doses greater than 500 kGy. The loss of selectivity could be attributed to the stochastic spatial distribution of native vacancies^70^ and those produced by elastic collisions involving energetic recoil electrons. Such vacancies within the basal plane of MoS_2_ would serve as reaction centers due to the introduction of vacant coordination sites.^23^ Hence, as reactions involving the products of adsorbate radiolysis proceed, radiolytic etching of the uppermost layer could emanate outwards, creating a “circle” in a similar fashion to the etching propagating inwards from the crystal edges, creating a “1 L-border.” Aside from the loss of selectivity, it should also be noted that upon irradiation with 1000 kGy: (1) the 1 L crystal is no longer visible, i.e., it is completely etched, (2) no circular holes are etched in the bottom layer of the 2 L crystal, and (3) the diameter of 1 L-border increases from ∼1.4 to ∼2 μm.

Prior to irradiation, the optical contrast is essentially invariant within the 1 L/2 L domains and across the substrate (Figure 3a). However, upon irradiation with an absorbed dose of 500 kGy, numerous circular features exhibiting distinct optical contrast are observed on the surface of the sample (Figure 3b). Moreover, the diameter and number of the circular features increase when irradiated with 1000 kGy (Figure 3c). Importantly, similar features have been observed on the surface of gamma-irradiated few-layer MoS_2_ films in the past.^38^ To assess whether these circular features are aggregates containing products of MoS_2_ radiolysis, a pristine SiO_2_/Si wafer was irradiated with a dose of 391 kGy in the absence of MoS_2_ crystals. Optical micrographs show that circular features with similar contrast and morphology are formed on the SiO_2_ surface after gamma irradiation (Figure S6, Supporting Information). Therefore, we conclude that the chemical composition of these features is likely to be organic and formed via radiolytic reactions involving adventitious carbon and/or adsorbed water. They are herein referred to as “carbonaceous aggregates.”

Figure 3d shows the Raman signal corresponding to the in-plane *E*_2g_^1^ normal vibrational mode of the pristine 1 L and 2 L MoS_2_ crystals visualized by optical microscopy. The frequency of the *E*_2g_^1^ mode is observed to blue shift when the thickness of the MoS_2_ crystal decreases from 2 L to 1 L, in accordance with the literature.^40^ The blue shift is attributed to an increase in the surface force constant caused by a slight charge redistribution due to the absence of an adjacent MoS_2_ layer.^71,72^ We utilized this characteristic spectroscopic distinction to further confirm the etching of MoS_2_ crystals under gamma irradiation. In particular, Raman spectroscopy maps are used in this work to evaluate the variation of the *E*_2g_^1^ frequency across the 1 L and 2 L MoS_2_ crystals after irradiation. Indeed, after irradiation with an absorbed dose of 500 kGy, the periphery of the 2 L crystal exhibits a significant blue shift of the *E*_2g_^1^ mode relative to the center of the 2 L domain (Figure 3e). Moreover, the average frequency of the in-plane *E*^′^ mode exhibited by the 1 L-border agrees well with the 1 L crystal present prior to irradiation. Hence, edge-selective radiolytic etching of 2 L MoS_2_ to yield 1 L domains has been unambiguously confirmed.

Upon irradiation with an absorbed dose of 1000 kGy, the Raman signal originating from the 1 L domain present prior to irradiation becomes negligible, thus confirming that the crystal has been etched completely (Figure 3f). The three circular features identified by optical microscopy exhibit a considerable blue shift of the *E*_2g_^1^ mode such that they agree well with the values observed within the surrounding 1 L-border, i.e., the loss of edge-selective radiolytic etching in 2 L crystals irradiated with the high absorbed doses is also confirmed.

Figure 3g shows the PL spectra of the pristine 2 L and 1 L MoS_2_ crystals visualized by optical microscopy. The typical PL spectrum of MoS_2_ consists of neutral A and B excitons observed at ∼1.90 and ∼ 2.05 eV, respectively.^6^ However, due to the deposition of the 2D MoS_2_ crystals onto SiO_2_, charge transfer from the substrate indicates that the A exciton exists as a negatively charged trion (A^–^) observed at ∼1.83 eV (Figure 3g).^73^ The optical properties of 2D MoS_2_ crystals are known to be strongly dependent on the crystal thickness.^74^ As mentioned earlier, an indirect–direct band gap transition is observed when 2 L MoS_2_ crystals are thinned to 1 L.^5^ The increased PL intensity of the 1 L crystal, relative to the 2 L domain, is attributed to the higher PL quantum efficiency of 1 L MoS_2_ due to its direct band gap (Figure 3g). Correspondingly, the PL intensity at the periphery of the 2 L crystal is increased relative to the internal region of the 2 L domain after 500 kGy irradiation (Figure 3h). This observation corroborates the conclusions reached via optical microscopy and Raman spectroscopy, outlined earlier, and further supports the hypothesis that the edge-selective radiolytic etching of the uppermost layer in 2 L MoS_2_ yields a 1 L domain at the periphery of the crystal, i.e., the formation of a 1 L-border. However, the PL intensity of the 1 L-border decreases significantly upon irradiation with an absorbed dose of 1000 kGy (Figure 3i). This is attributed to the formation of midgap states, introduced by defects, which increase the number of nonradiative decay pathways and lead to the observed reduction in the PL quantum yield.^23^ Moreover, it should be noted that radiolytic etching is not limited to 2 L crystals yielding 1 L domains. Etching of 3 L and 4 L crystals has also been observed and investigated by correlative optical microscopy and Raman/PL spectroscopy maps; see the discussion and Figures S7–S11 in the Supporting Information. In addition, the full Raman spectra of 1 L crystals (pristine and 500 kGy) and the 1 L border region (500 kGy) are available in Figure S12, Supporting Information.

### 3.3 Radiation-Induced Doping of MoS_2_

**Figure 4:**
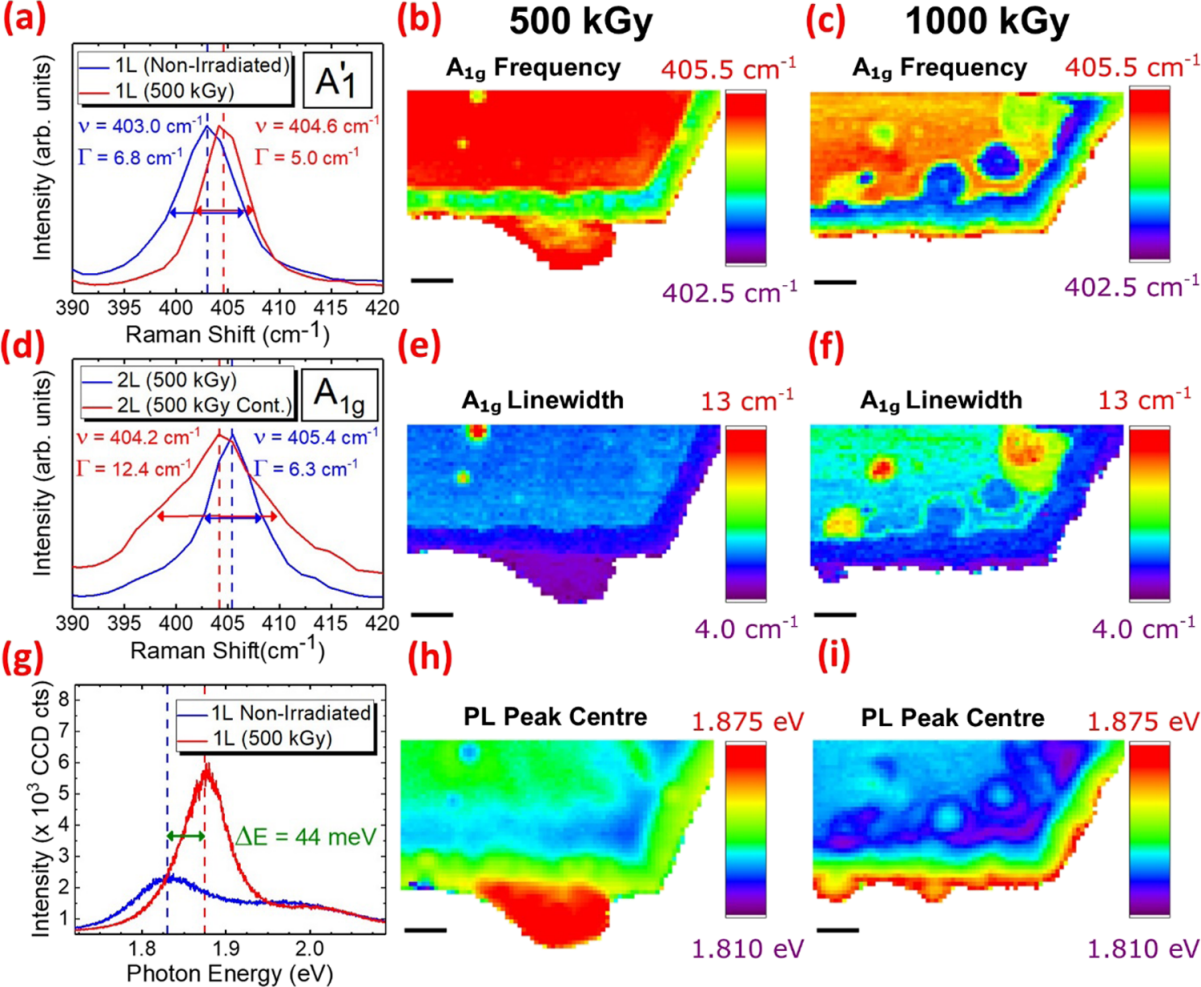
Top row: (a) Raman spectra of the 1 L MoS_2_ crystal domain prior to irradiation (blue) and after irradiation with an absorbed dose of 500 kGy (red) showing an increase in the frequency (*υ*) and reduction in the line width (Γ) of the out-of-plane *A*_1_^′^ mode. Correlative Raman maps of the 1 L and 2 L domains visible in the optical micrographs showing the variation of the *A*_1g_ frequency across the crystals after irradiation with absorbed doses of (b) 500 kGy and (c) 1000 kGy. Middle row: (d) Raman spectra of the 2 L domain after irradiation with an absorbed dose of 500 kGy showing a decrease in the *υ* and an increase in the Γ of the *A*_1g_ mode when the crystal is contaminated (Cont.) with carbonaceous aggregates (red) relative to the uncontaminated 2 L region (blue). Correlative Raman maps of the 1 L and 2 L domains visible in the optical micrographs showing the variation of the *A*_1g_ line width across the crystals after irradiation with absorbed doses of (e) 500 kGy and (f) 1000 kGy. Bottom row: (g) Photoluminescence (PL) spectra of the 1 L MoS_2_ domain showing a blue shift and an increase in the PL intensity when the crystal is irradiated with an absorbed dose of 500 kGy (red) relative to the same region prior to irradiation (blue). Correlative PL maps of the 1 L and 2 L domains visible in the optical micrographs showing the variation of the PL peak center across the crystals after irradiation with absorbed doses of (h) 500 kGy and (i) 1000 kGy. All scale bars correspond to a length of 2 μm.

In addition to the changes in the chemical composition and morphology of the crystals under gamma irradiation, correlative optical microscopy and Raman and PL spectroscopy maps have been used to gain insights into the doping mechanisms of 2D MoS_2_ irradiated within the highly absorbed dose regime (Figure 4). The maps shown in Figure 4 are obtained from the same 1 L and 2 L crystals shown in the optical micrographs in Figure 3, after irradiation with an absorbed dose of 500 kGy (Figures 3b and 4b,e,h) and 1000 kGy (Figures 3c and 4c,f,i). The optical micrographs can be utilized to aid spatial correlation between crystal domains and changes in their vibrational and optical properties.

Figure 4a shows the Raman signal of the pristine 1 L MoS_2_ domain corresponding to the out-of-plane *A*_1_^′^ normal vibrational mode, which exhibits a frequency and line width of 403.0 and 6.8 cm^–1^, respectively. Upon irradiation with an absorbed dose of 500 kGy, the *A*_1_^′^ mode of the 1 L crystal exhibits a 1.6 cm^–1^ blue shift, while the line width decreases by 1.8 cm^–1^. The frequency and line width of the *A*_1_^′^ mode in 1 L MoS_2_ are known to be strongly modulated by doping,^75^ and the observed changes indicate that 1 L MoS_2_ becomes p-doped^23^ after 500 kGy irradiation.

Regarding the mechanism of gamma radiation-induced p-doping, the efficiency of charge transfer between oxygen and 1 L MoS_2_ increases when O_2_ is adsorbed onto S vacancies, as opposed to the pristine basal plane of the crystal.^76^ Such vacancies can be introduced into the lattice via elastic collisions involving energetic recoil electrons. When O_2_ adsorbs onto radiation-induced vacancies, charge-transfer interactions lead to a lowering of the Fermi level, i.e., MoS_2_ becomes p-doped.

Moreover, the out-of-plane *A*_1_^′^ phonons retain the symmetry of the MoS_2_ lattice; hence, they couple strongly with electrons.^75^ Therefore, as p-doping is expected to decrease the amount of electrons occupying antibonding states in the conduction band of 1 L MoS_2_, this results in an increase in the force constant of the out-of-plane vibration, i.e., the *A*_1_^′^ mode blue shifts. Regarding the reduction in the line width of the *A*_1_^′^ mode, this is a direct consequence of the electron–phonon coupling, i.e., the line width of a Raman signal is influenced by both the lifetime of a phonon and how strongly it couples to electrons.^75^ Indeed, p-type changes to the Raman spectrum of defective 2D MoS_2_ have been observed previously and attributed to charge-transfer interactions involving adsorbed oxygen.^23^

Figure 4b shows the variation of the *A*_1g_ frequency across the 1 L and 2 L crystals after irradiation with an absorbed dose of 500 kGy. The *A*_1_^′^ frequency of the 1 L-border produced by edge-selective radiolytic etching is blue shifted by approximately 1 cm^–1^ relative to the value measured for pristine 1 L MoS_2_ prior to irradiation. This is in contrast to the p-doped 1 L domain present prior to irradiation, i.e., not produced by etching, which exhibits a more pronounced 1.6 cm^–1^ shift, as previously outlined (Figure 4a). Indeed, upon irradiation with an absorbed dose of 1000 kGy, the *A*_1_^′^ frequency at the periphery of the 1 L-border remains blue shifted by approximately 1 cm^–1^ (Figure 4c). However, the blue shift within the central region of the 1 L-border is <1 cm^–1^, i.e., not statistically significant. Therefore, a possibility of p-doping of 1 L MoS_2_ domains created by radiolytic etching would require further investigation.

Figure 4d shows the Raman signal corresponding to the *A*_1g_ mode of the 2 L MoS_2_ crystal irradiated with an absorbed dose of 500 kGy. Two distinct Raman shift values are observed in the spectra. The first one is acquired from a surface region of the 2 L crystal, which is contaminated with carbonaceous aggregates, while the other is acquired from a neighboring uncontaminated region. A significant red shift and line width increase of the *A*_1g_ mode is observed when carbonaceous aggregates are adsorbed on 2 L MoS_2_ crystals. Indeed, the spatial distribution of the aggregates, observed in the optical micrographs of MoS_2_ crystals irradiated with absorbed doses of 500 kGy (Figure 3b) and 1000 kGy (Figure 3c), can be correlated with a red shift and increased line width of the *A*_1g_ mode relative to the neighboring uncontaminated regions: 500 kGy, Figure 4b,e and 1000 kGy, Figure 4c,f. The observed changes are qualitatively consistent with n-doping of MoS_2_.^75^ The magnitude of the observed red shift is smaller than those afforded by the in situ measurements of 1 L MoS_2_ field-effect transistors.^75^ However, the adsorption of the carbonaceous aggregates is likely to increase the effective restoring forces acting on S atoms vibrating out-of-plane, thus leading to a reduction in the magnitude of the anticipated *A*_1g_ redshift.

To gain insights into the effects of radiation-induced p-doping on the optical properties of 1 L MoS_2_ crystals, Figure 4g shows the PL spectrum of the 1 L domain prior to and after irradiation with an absorbed dose of 500 kGy. A significant increase in the PL intensity and concurrent ∼44 meV blue shift of the A^–^ trion are observed upon irradiation. The blue shift of the negative A^–^ trion PL signal is such that its value increases from ∼1.83 to ∼1.87 eV, i.e., the A^–^ trion dissociates into the neutral A exciton at a higher energy.^77^ We propose that the dissociation of the A^–^ trion could be facilitated by charge-transfer interactions involving occupied conduction band states in MoS_2_ and O_2_ molecules adsorbed onto radiation-induced vacancies, akin to the mechanism responsible for the blue shift and line width increase of the *A*_1_^′^ mode discussed previously. Equivalently, one could state that gamma radiation-induced p-doping of 1 L MoS_2_ simply suppresses A^–^ trion formation. Moreover, as trions possess a greater variety of nonradiative decay pathways with respect to excitons,^78^ the significant increase in the PL signal observed (Figure 4g) can also be attributed to the dissociation/suppression of A^–^ trions in gamma-irradiated 1 L crystals. The optical and out-of-plane vibrational properties of 1 L crystals irradiated with an absorbed dose of 500 kGy (Figure 4a,g) are consistent with those reported for 1 L MoS_2_ in which p-doping is introduced via charge-transfer interactions involving either halogenated solvents^79^ or O_2_ adsorbed onto heavy ion-irradiated crystals.^23^

Figure 4h shows the variation in the PL peak center across the 1 and 2 L crystals after irradiation with an absorbed dose of 500 kGy. It can be observed that the PL emission corresponding to the neutral A exciton at ∼1.87 eV is strongly localized within the 1 L domain that was present prior to irradiation and correlates with the ∼1.5 cm^–1^ blue shift of the *A*_1_^′^ mode (Figure 4b), unambiguously confirming the radiation-induced p-doping of 1 L MoS_2_. However, the peak center of the PL signal originating from within the 1 L-border and 2 L crystal domains varies by just ±10 meV from the ∼1.83 eV value exhibited by A^–^ trion emission, measured prior to irradiation. Moreover, despite the significant blue shift of the PL peak center at the periphery of the 1 L-border after irradiation with an absorbed dose of 1000 kGy (Figure 4i), this shift is not statistically significant within the internal region of the 1 L-border, akin to the <1 cm^–1^ blue shift of the *A*_1_^′^ mode discussed earlier (Figure 4c). Hence, radiation-induced p-doping in 1 L MoS_2_ domains created by edge-selective radiolytic etching cannot be confirmed definitively.

To assess the influence of crystal morphology on the gamma radiation-induced p-doping of MoS_2_, a polycrystalline 1 L film produced by CVD was irradiated with an absorbed dose of 40 kGy, and Raman and PL maps were acquired pre- and post-irradiation. Statistical analysis of the maps suggests that the 1 L film becomes more defective and p-doped upon irradiation; these results are shown in Figure S13, Supporting Information.

## 4 Conclusions

The effect of high-dose gamma irradiation on the chemical composition, morphology, and vibrational and optical properties of MoS_2_ crystals under ambient conditions has been systematically evaluated. The deconvolution of XPS spectra as a function of radiation exposure shows that the surface of the MoS_2_ crystal undergoes significant oxidation via a series of intermediates to ultimately yield Mo^VI^S*_y_*O*_x_* and sulfate species. Oxidation of MoS_2_ is driven by reactions involving the products of adsorbate radiolysis such as water and adventitious carbon. Moreover, edge-selective radiolytic etching of the uppermost layer of MoS_2_ crystals is observed after irradiation with an absorbed dose of 500 kGy, evidenced by correlative optical microscopy and Raman and PL spectroscopy. The observed etching is facilitated by the higher reactivity of active edge sites and the lower rate of reaction between the products of adsorbate radiolysis and MoS_2_ layers further from the interface. A blue shift and a line width decrease of the out-of-plane *A*_1_^′^ vibrational mode are observed upon irradiation of 1 L MoS_2_ with an absorbed dose of 500 kGy and are attributed to p-doping. The mechanism of p-doping is expected to involve the adsorption of O_2_ molecules onto radiation-induced vacancies and subsequent charge-transfer interactions. Consequently, the p-doping of gamma-irradiated 1 L MoS_2_ significantly alters the optical properties of the crystal as A^–^ trions are observed to dissociate into neutral A excitons. In addition to the oxidation, etching, and doping of MoS_2_ crystals observed at lower doses, upon irradiation with an absorbed dose of 1000 kGy, monolayer MoS_2_ crystals are completely etched from the substrate. Hence, our results provide fundamental insights into key radiation damage mechanisms occurring at the MoS_2_ crystal surface as well as the threshold of the material’s radiation hardness in the ≤1000 kGy range. Additionally, we demonstrate that the radiolytic degradation of MoS_2_ is accelerated in the presence of adsorbed water due to the production of reactive radical species.

We conclude that gamma irradiation of MoS_2_ results in the adverse changes in the physical and chemical properties, which would lead to a gradual degradation of MoS_2_-based device performances. For example, vacancies produced by the radiation-induced displacement damage will introduce defect states within the band gap of MoS_2_. In FETs, such vacancies would act as scattering centers for charge carriers, thus reducing carrier mobility, while radiolytic oxidation would change the chemical composition of MoS_2_, resulting in deviation from the expected current–voltage characteristics of a pristine MoS_2_ device. In optoelectronic devices, radiation-driven doping would change the intensity of PL, i.e., alter the quantum efficiency of the PL process, and PL emission energy of the device, i.e., alter the wavelength of emission.

We summarize that, owing to their appreciable radiation stability, 2D MoS_2_-based devices are promising candidates for a variety of demanding nuclear and space applications. However, appropriate consideration must be given to protective shielding and required device operation times as well as to the presence of common adsorbates, such as water, on the surface of MoS_2_ during radiation exposure.

## Supporting Information Available

Compton scattering calculations; X-ray photoelectron spectroscopy of humidity-conditioned MoS_2_ crystals; X-ray photoelectron spectroscopy of polycrystalline monolayer MoS_2_ films; optical microscopy of SiO_2_/Si substrates; correlative optical microscopy and Raman/PL mapping of few-layer MoS_2_ crystals; full Raman spectra of 1 L MoS_2_ crystals and 1 L border region after 500 kGy irradiation; statistical analysis of Raman/PL maps of polycrystalline monolayer MoS_2_ films (PDF)
