# Supplementary material for: Gamma Radiation-Induced Oxidation, Doping, and Etching of Two-Dimensional MoS2 Crystals
Source: J Phys Chem C Nanomater Interfaces. 2021 Feb 10;125(7):4211–22. doi: 10.1021/acs.jpcc.0c10095 (PMC8025684; doi:10.1021/acs.jpcc.0c10095)
Supplement: Supplementary file 1 — jp0c10095_si_001.pdf [file jp0c10095_si_001.pdf]

Supporting Information

Gamma Radiation-Induced Oxidation, Doping and Etching of Two-Dimensional MoS_2 Crystals

*Liam H. Isherwood [†, §], Gursharanpreet Athwal [†], Ben F. Spencer [‡], Cinzia Casiraghi [†], Aliaksandr Baidak [†, §] **

[†] Department of Chemistry, School of Natural Sciences, University of Manchester, Manchester, M13 9PL, UK
[§] Dalton Cumbrian Facility, Dalton Nuclear Institute, University of Manchester, Cumbria, CA24 3HA, UK
[‡] Department of Materials, School of Natural Sciences, University of Manchester, Manchester, M13 9PL, UK

1. Compton Scattering Calculations

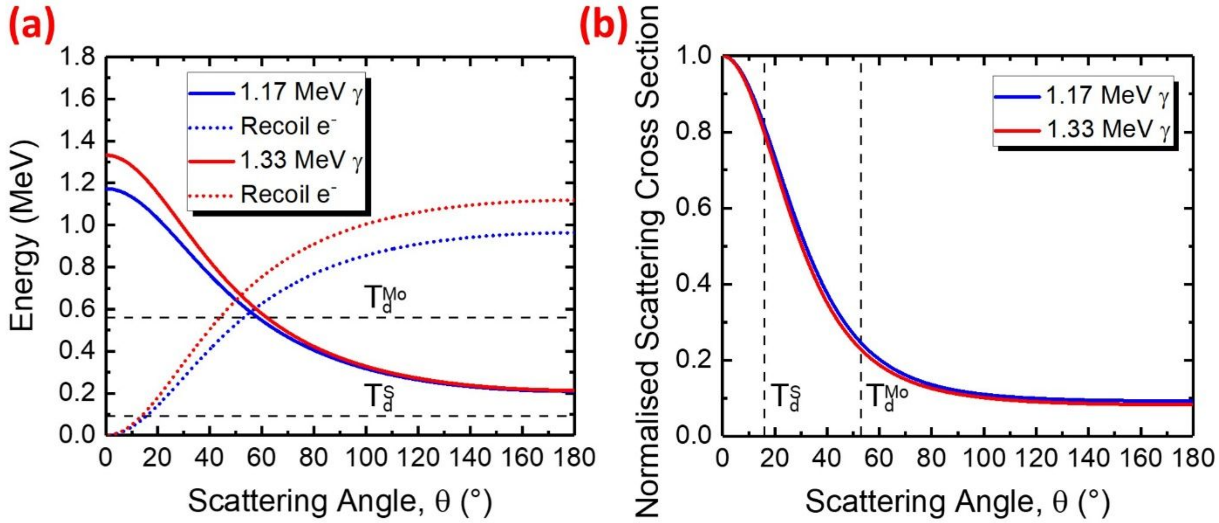

Figure S1: **(a)** Scattered photon energy (solid curve) and kinetic energy of recoil electron (dotted curve) as a function of scattering angle for 1.17 MeV (blue) and 1.33 MeV (red) photons. Reference lines correspond to the recoil electron kinetic energies required for displacement of Mo (T_d^{Mo}) or S (T_d^S) atoms from their lattice sites in MoS_2 crystals (i.e. T_d^{Mo} = 560 keV and T_d^S = 90 keV). **(b)** Normalised differential cross section for Compton scattering of 1.17 MeV (blue curve) and 1.33 MeV (red curve) photons as a function of scattering angle. Reference lines depict the scattering angles required to produce a recoil electron with a kinetic energy corresponding to T_d^{Mo} or T_d^S (i.e. T_d^{Mo} = 53° and T_d^S = 16° for 1.17 MeV photons).

The ^{60}Co source used during gamma irradiation of MoS_2 crystals undergoes negatron emission to yield a nuclear excited state of ^{60}Ni, which relaxes to the ground state *via* emission of two gamma photons with energies of 1.17 MeV and 1.33 MeV:

$$^{60}_{27}Co \rightarrow ^{60}_{28}{}^{*}Ni + \beta^{-} + \bar{\upsilon}_e$$

$$^{60}_{28}{}^{*}Ni \rightarrow ^{60}_{28}Ni + 2\gamma$$

For gamma photons in this energy regime, their principle interaction mechanism with matter is Compton scattering i.e. inelastic scattering by valence electrons. The change in the wavelength (energy) of the incident photon, $\Delta\lambda$, can be calculated by:

$$\lambda' - \lambda = \Delta\lambda = \frac{h}{m_e c}(1 - \cos\theta)$$

Where λ' and λ represent the wavelength of the scattered and incident photon, respectively, h is the Planck constant, m_e is the electron rest mass, c is the speed of light and θ is the scattering angle. As valence electrons are weakly bound and the energy of the incident photons produced by ^{60}Co decay is known, the energy of the scattered photon and kinetic energy of the recoil electron can be calculated as a function of the scattering angle (**Figure S1(a)**). The inverse relationship between the energies of scattered photon and recoil electron is easily conceptualised as maximum energy transfer will occur during a head-on collision i.e. when $\theta = 180°$.

Unlike photons, as the energetic recoil electrons have a finite mass, they can engage in elastic collisions with the Mo and S atoms in the lattice. Hence, vacancies can be produced in MoS_2 crystals under gamma irradiation, providing that the kinetic energy transferred to the Mo or S recoil atom is greater than its threshold displacement energy. The threshold displacement energies of Mo and S atoms in MoS_2 are calculated to be 20 eV and 6.9 eV, respectively, corresponding to required electron kinetic energies of 560 keV (T_d^{Mo}) and 90 keV (T_d^{S}).[1] Hence, Figure S1(a) shows that *via* Compton scattering, ^{60}Co decay produces recoil electrons with sufficient kinetic energy to create Mo and S vacancies in MoS_2 crystals.

However, the cross section for Compton scattering depends strongly on the scattering angle i.e. the probability of producing a recoil electron with a given kinetic energy is characterised by a distribution. Therefore, the normalised cross section for Compton scattering, as a function of θ, provides insights into

the relative yields of Mo/S vacancies produced in MoS_2 crystals under ^{60}Co gamma irradiation and can be calculated using the Klein-Nishina formula:

$$\frac{d\sigma}{d\Omega} = \frac{{r_e}^2}{2}\left(\frac{h\nu'}{h\nu}\right)^2\left(\frac{h\nu'}{h\nu} + \frac{h\nu}{h\nu'} - \sin^2\theta\right)$$

Where $\frac{d\sigma}{d\Omega}$ represents the differential scattering cross section per unit of solid angle, r_e is the classical electron radius, ν' and ν represent the frequencies of the scattered and initial photons, respecetively, h is the Planck constant and θ is the scattering angle (**Figure S1(b)**). For 1.17 MeV photons, scattering angles of 53° and 16° produce recoil electrons with energies corresponding to T_d^{Mo} and T_d^{S}, respectively. Therefore, Figure S1(b) shows that S vacancy formation is significantly more likely compared with Mo vacancies. The normalised scattering cross section for 1.17 MeV photons is integrated between 0° - 180° and 16° - 180°, following which, the ratio of the calculated areas suggests that ~68% of all recoil electrons possess sufficient kinetic energy to displace S atoms.

2. X-ray Photoelectron Spectroscopy of Humidity-Conditioned MoS_2 Crystals

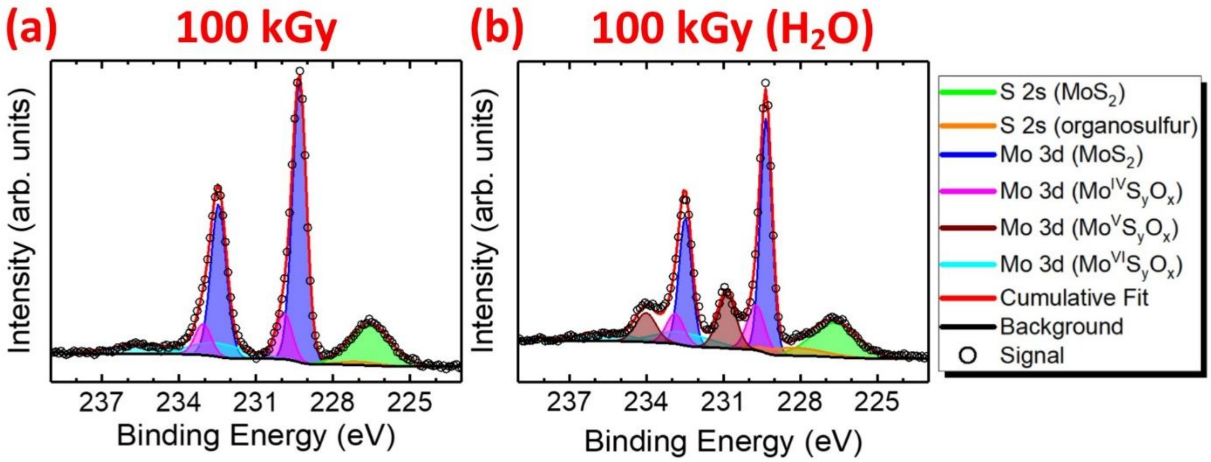

Figure S2: Deconvoluted Mo 3d x-ray photoelectron spectra of MoS_2 crystals after irradiation to an absorbed dose of 100 kGy. MoS_2 crystals are micromechanically exfoliated and deposited onto SiO_2 wafers, following which, they are stored either **(a)** under ambient conditions or **(b)** conditioned for 5 days at 95% relative humidity prior to irradiation.

As discussed in the main text, it is hypothesised that $Mo^{V}S_yO_x$ intermediates may be the sole product of reactions between MoS_2 surface atoms and either water radiolysis products or gaseous O_2. In order to investigate this, two samples were produced and irradiated to an absorbed dose of 100 kGy. Both samples consist of MoS_2 crystals, produced by micromechanical exfoliation (MME), deposited on SiO_2/Si substrates. However, one sample was stored under ambient conditions prior to irradiation whilst the other was stored in a chamber at 95% relative humidity for 5 days prior to irradiation. Such conditioning significantly promotes adsorption of H_2O onto the surface of the crystals. Hence, the samples are denoted 100 kGy and 100 kGy (H_2O), respectively.

Figure S2 shows the deconvoluted Mo 3d x-ray photoelectron spectra of the 100 kGy and 100 kGy (H_2O) samples. The 100 kGy sample exhibits one singlet and three doublets corresponding to the S^{2-}, $Mo^{IV}S_2$, $Mo^{IV}S_yO_x$ and $Mo^{VI}S_yO_x$ environments (**Figure S2(a)**), as outlined in the main text. The integrated intensities of the aforementioned photoelectron lines are very similar in the Mo 3d spectrum of the 100 kGy (H_2O) sample (**Figure S2(b)**). However, a new doublet is observed in which the Mo $3d_{5/2}$ and Mo $3d_{3/2}$ photoelectron lines exhibit binding energies of 230.9 eV and 234.0 eV, respectively. The binding energies of these Mo 3d core level electrons agree well with those observed for Mo^{V} centres in oxysulfide thin films.[2] Hence, the doublet is attributed to the radiolytic oxidation to yield Mo^{V} centres with a mixed bonding environment i.e. formation of $Mo^{V}S_yO_x$ species. It should be noted that $Mo^{V}S_yO_x$ formation is observed in samples stored under ambient conditions and irradiated to an absorbed dose of 500 kGy (**Figure 2(c)**, main text) i.e. five times higher than the dose administered to the 100 kGy (H_2O) sample. Hence, Figure S2(b) definitively proves that MoS_2 oxidation is driven to a large extent by the products of water radiolysis. Moreover, as oxidation of MoS_2 *via* heavy ion irradiation and O_2 adsorption does not seem to yield $Mo^{V}S_yO_x$,[3,4] **Figure S2(b)** suggests that $Mo^{V}S_yO_x$ intermediates are the sole product of reactions between MoS_2 surface atoms and the products of water radiolysis.

Relative to the 100 kGy sample (Figure S2(a)), the structure of the S 2s signal in the 100 kGy (H_2O) sample exhibits notable asymmetry at higher binding energy (**Figure S2(b)**). Deconvolution of the signal suggests that a higher concentration of organosulfur species are produced during gamma irradiation of MoS_2 crystals in the presence of elevated levels of adsorbed water.

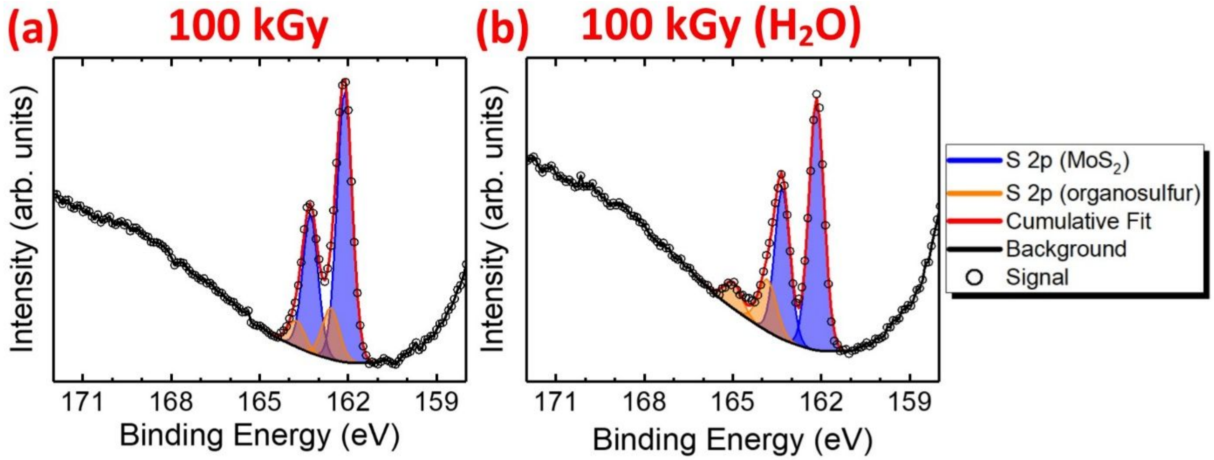

Figure S3: Deconvoluted S 2p x-ray photoelectron spectra of MoS_2 crystals after irradiation to an absorbed dose of 100 kGy. MoS_2 crystals are micromechanically exfoliated and deposited onto SiO_2 wafers, following which, they are stored either **(a)** under ambient conditions or **(b)** conditioned for 5 days at 95% relative humidity prior to irradiation.

The deconvoluted S 2p x-ray photoelectron spectrum of the 100 kGy sample (**Figure S3(a)**) exhibits two doublets associated to S^{2-} and organosulfur environments, as described in the main text. However, in the 100 kGy (H_2O) sample, the binding energy of the organosulfur signal is ~ 1.2 eV higher such that the S $2p_{3/2}$ and S $2p_{1/2}$ photoelectron lines exhibit values of 163.8 eV and 165.0 eV, respectively (**Figure S3(b)**). This observation is in agreement with the more pronounced asymmetry of the S 2s signal in the Mo 3d spectrum of the 100 kGy (H_2O) sample (Figure S2(b)).

Moreover, similarly to the presence of $Mo^{V}S_yO_x$ species in the Mo 3d spectrum, the binding energies of the organosulfur species in the S 2p spectrum of the 100 kGy (H_2O) sample agree well with those exhibited after 500 kGy irradiation of MoS_2 crystals stored under ambient conditions (**Figure 1(c)**, main text). Hence, the Mo 3d (Figure S2) and S 2p (Figure S3) x-ray photoelectron spectra provide conclusive

evidence that the products of water radiolysis directly influence and accelerate the oxidation of MoS_2 crystals. This is further evidenced by the relative concentration of Mo (**Table 1**) and S (**Table 2**) chemical species present in the 100 kGy and 100 kGy (H_2O) samples.

Species/ Sample	MoS_2 [%]	$Mo^{IV}S_yO_x$ [%]	$Mo^{V}S_yO_x$ [%]	$Mo^{VI}S_yO_x$ [%]
**100 kGy**	**71.49**	**14.18**	**0**	**14.33**
**100 kGy (H_2O)**	**51.90**	**15.51**	**18.62**	**13.97**

Table 1. Percentage of Mo atoms in a given chemical state, relative to the total integrated intensity of the Mo 3d photoelectron signal, for both 100 kGy and 100 kGy (H_2O) samples.

Species/ Sample	Metal Sulfide [%]	Organosulfur [%]
**100 kGy**	**92.26**	**7.74**
**100 kGy (H_2O)**	**81.07**	**18.93**

Table 2. Percentage of S atoms in a given chemical state, relative to the total integrated intensity of the S 2p photoelectron signal, for both 100 kGy and 100 kGy (H_2O) samples.

3. X-ray Photoelectron Spectroscopy of Polycrystalline Monolayer MoS_2 Films

The S 2p x-ray photoelectron spectrum of the pristine polycrystalline monolayer (1L) MoS_2 film, produced by chemical vapour deposition (CVD), exhibits a doublet corresponding to the S^{2-} environment in which the S $2p_{3/2}$ and S $2p_{1/2}$ photoelectron lines possess binding energies of 162.4 eV and 163.5 eV, respectively (**Figure S4(a)**). These values are ~ 0.6 eV higher than those observed for MoS_2 single crystals deposited *via* MME (Figure 1(a), main text). This is in agreement with the common observation of n-doping in MoS_2 crystals produced by CVD.[5] Moreover, a second doublet is observed in the S 2p spectrum of pristine 1L MoS_2, produced by CVD¸ in which the S $2p_{3/2}$ and S $2p_{1/2}$ photoelectron lines exhibit binding energies of 164.3 eV and 165.5 eV, respectively. These values do not agree with those reported for sulfoxide compounds,[6,7] aliphatic sulfides[7] or thiols.[8] Moreover, formation of these species is highly unlikely as CVD process is carried out under inert atmosphere and, obviously, the pristine sample has not

yet been exposed to gamma radiation. However, the binding energy of the second doublet agrees well with the values reported for the S^0 environment of elemental sulfur[9] which could be deposited during sulfidation step of CVD. However, as the sample was purchased from a commercial retailer, the exact deposition protocol is unknown.

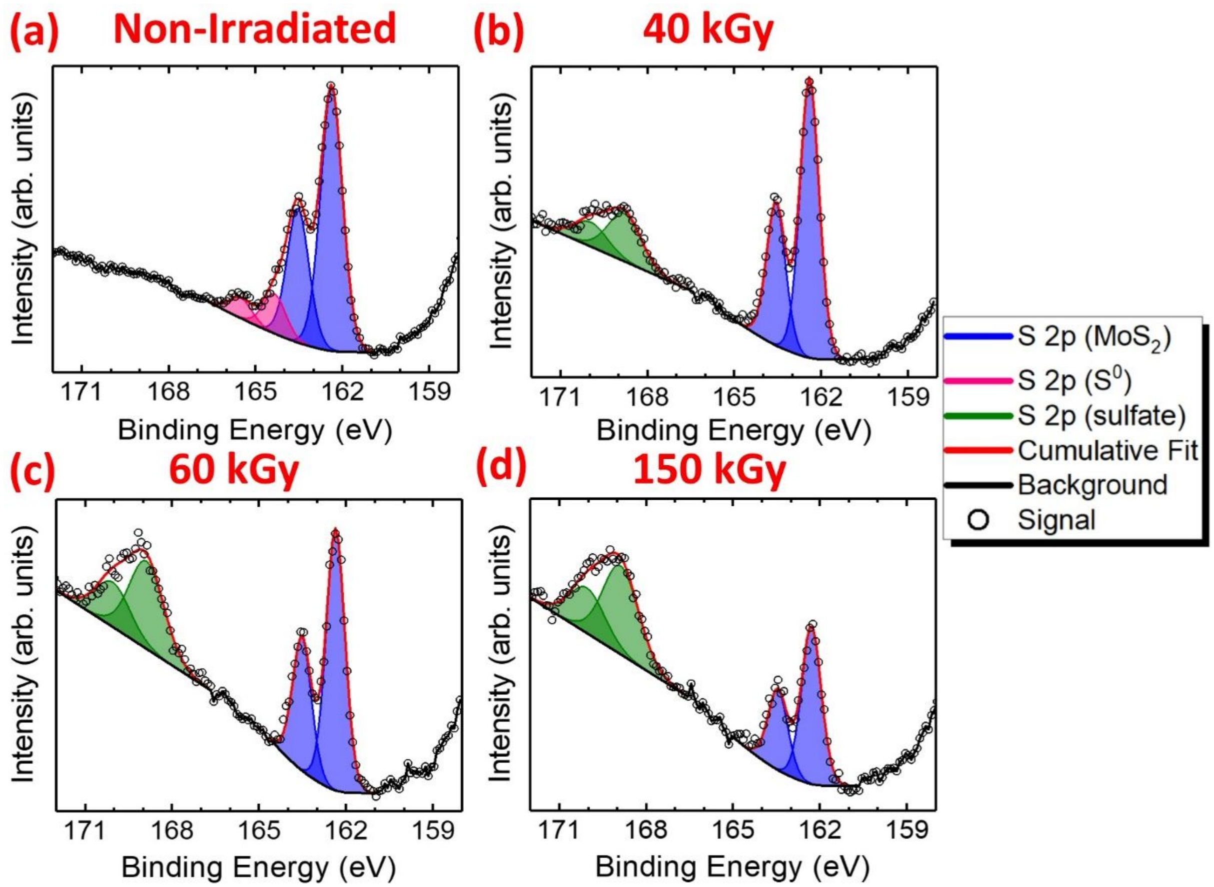

Figure S4: Deconvoluted S 2p x-ray photoelectron spectra of polycrystalline monolayer MoS_2 films, produced by chemical vapour deposition, **(a)** prior to irradiation and after irradiation to absorbed doses of **(b)** 40 kGy, **(c)** 60 kGy and **(d)** 150 kGy.

Upon irradiation to an absorbed dose of 40 kGy the signal attributed to the S^0 environment of elemental sulfur is no longer observed (**Figure S4(b)**). It is likely that the elemental sulfur is oxidised to yield sulfate species *via* reactions involving the products of water radiolysis.[10] Indeed, a new doublet is observed in which the S $2p_{3/2}$ and S $2p_{1/2}$ photoelectron lines exhibit binding energies of 168.8 eV and 169.9 eV, respectively, which agree well with those reported for sulfate compounds.[11]

At higher absorbed doses of 60 kGy (**Figure S4(c)**) and 150 kGy (**Figure S4(d)**), the integrated intensity of the sulfate photoelectron signal increases significantly relative to the S^{2-} environment. As there is no elemental sulfur present after irradiation to an absorbed dose of 40 kGy (Figure S4(b)), this suggests that the increase in the concentration of sulfate species at higher adsorbed doses is due to radiolytic oxidation of the 1L MoS_2 film *via* reactions involving the products of water radiolysis.

As detailed in the main text, the radiolytic oxidation of S atoms in MoS_2 crystals deposited by MME progresses *via* the formation of organosulfur intermediates to eventually yield sulfate species at absorbed doses of $\geq$ 500 kGy (Figure 1, main text). Therefore, upon irradiation of 1L MoS_2 produced by CVD to absorbed doses between 40 kGy and 150 kGy, the absence of organosulfur intermediates and high concentrations of sulfate species suggests that the rate of radiolytic oxidation is considerably higher for samples produced by CVD with respect to MME.

This may be rationalised by the morphology of the material produced by CVD. As the 1L film is polycrystalline, the fraction of atoms at grain boundaries and crystal edges verses those within the basal plane is likely to be significantly higher with respect to MoS_2 single crystals deposited by MME. Therefore, similarly to the mechanism of edge-selective radiolytic etching, discussed in the main text, the radiolytic oxidation of polycrystalline 1L MoS_2 produced by CVD is likely proceed at lower absorbed doses due to the high reactivity of grain boundaries and active edge sites.[12–14]

Prior to irradiation, the Mo 3d x-ray photoelectron spectrum of the polycrystalline 1L MoS_2 films produced by CVD contains three doublets and two singlets (**Figure S5(a)**). The S 2s singlet exhibiting a binding energy of 228.8 eV corresponds to the S^0 environment of elemental sulfur as rationalised earlier. The photoelectron lines corresponding to the Mo^{IV} and S^{2-} environments of MoS_2 are ~ 0.6 eV higher than those observed for MoS_2 single crystals deposited *via* MME (Figure 2(a), main text) due to the inherent n-doping of MoS_2 crystals produced by CVD.[5]

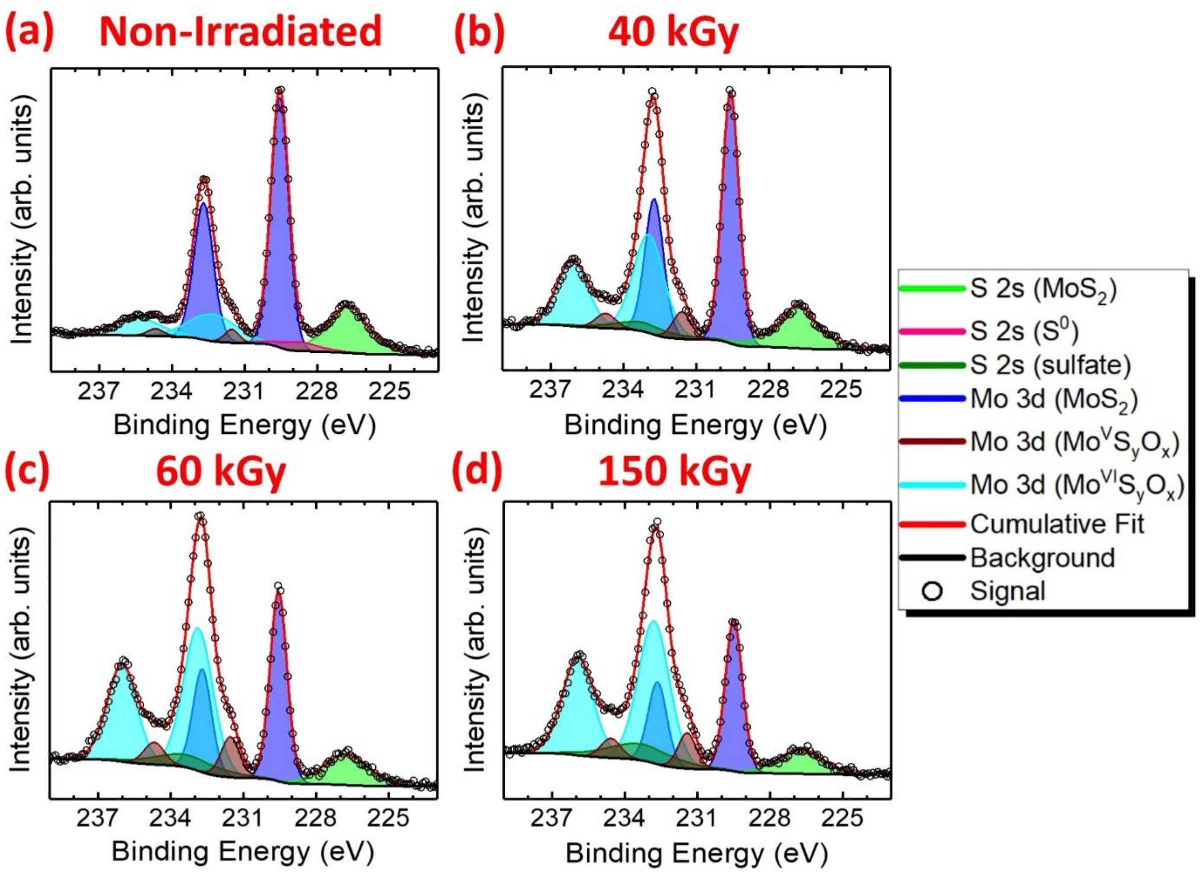

Figure S5: Deconvoluted Mo 3d x-ray photoelectron spectra of polycrystalline monolayer MoS_2 films, produced by chemical vapour deposition, **(a)** prior to irradiation and after irradiation to absorbed doses of **(b)** 40 kGy, **(c)** 60 kGy and **(d)** 150 kGy.

The doublet exhibiting Mo $3d_{5/2}$ and Mo $3d_{3/2}$ binding energies of 232.3 eV and 235.5 eV, respectively, is attributed to the presence of Mo^{VI} centres.[15,16] Indeed, Mo^{VI} species are ubiquitous even in high quality 1L MoS_2 crystals produced by CVD.[17] However, as the stoichiometry of the Mo^{VI} bonding environment is unknown, they are given the general formula $Mo^{VI}S_yO_x$. Moreover, a third doublet is observed in which the Mo $3d_{5/2}$ and Mo $3d_{3/2}$ photoelectron lines exhibit binding energies of 231.5 eV and 234.7 eV, respectively; this signal is attributed to a small concentration of $Mo^{V}S_yO_x$ species.

Upon irradiation to absorbed doses of 40 kGy (**Figure S5(b)**), 60 kGy (**Figure S5(c)**) and 150 kGy (**Figure S5(d)**), the integrated intensities of the $Mo^{VI}S_yO_x$ and $Mo^{V}S_yO_x$ signals increase whilst the MoS_2 (Mo^{4+} and S^{2-}) signals decrease as a function of radiation exposure. This agrees with the dependencies of the $SO_4{}^{2-}$ and S^{2-} signals in the S 2p spectra (Figure S4). Also akin to the S 2p spectra, the S^0 environment

of elemental sulfur is not observed in the Mo 3d spectrum of the 1L film after irradiation to an absorbed dose of 40 kGy (Figure S5(b)). Accordingly, a considerable increase in the S 2s singlet at 233.3 eV, corresponding to the sulfate environment, is observed.

These findings show that the rate at which the 1L film becomes oxidised, to produce $Mo^{VI}S_yO_x$ and sulfate species, is the higher for CVD samples relative to MoS_2 single crystals deposited by MME. As outlined earlier, this could be attributed to the polycrystalline nature of 1L MoS_2 films produced by CVD i.e. more atoms are located at grain boundaries and crystal edges. Moreover, the considerably higher concentration of $Mo^{VI}S_yO_x$ and sulfate species in polycrystalline 1L films could be attributed to its large surface area to volume ratio. For instance, in few-layer crystals, such as those deposited by MME, the rate of reactions between the products of water radiolysis/oxygen and buried MoS_2 layers far from the crystal-atmosphere interface are expected to be lower leading to slower oxidation of the MoS_2 crystal. Conversely, in 1L MoS_2 films produced by CVD, a higher fraction of atoms will reside at the surface of the crystal, leading to faster oxidation of the nanostructure.

4. Optical Microscopy of SiO_2/Si Substrates

In order to assess whether the circular features formed during gamma irradiation of MoS_2/SiO_2/Si samples are a product of MoS_2 radiolysis, a freshly cleaned SiO_2/Si wafer was irradiated to an absorbed dose of 391 kGy in the absence of MoS_2 crystals. Prior to irradiation, the SiO_2/Si wafer was sonicated for 20 minutes in a solution of propan-2-ol (99.9%, Sigma Aldrich). The wafer was sonicated for a further 10 minutes in acetone (99.9 %, Sigma Aldrich), following which, residual solvent was removed using N_2 airflow. Upon drying, the wafer was crimp-sealed inside a borosilicate vial and exposed to gamma radiation.

"Circular features", akin to those reported by Ozden *et al* on the surface of gamma irradiated 2D MoS_2 films produced by CVD,[28] were observed on the SiO_2 surface of the wafer after irradiation to an absorbed dose of 391 kGy (**Figure S6**). Therefore, the circular features are not a product of MoS_2 radiolysis, rather,

they are likely to be carbonaceous aggregates formed *via* radiolytic reactions involving adventitious carbon which is ubiquitous on all surfaces exposed to ambient conditions.[29]

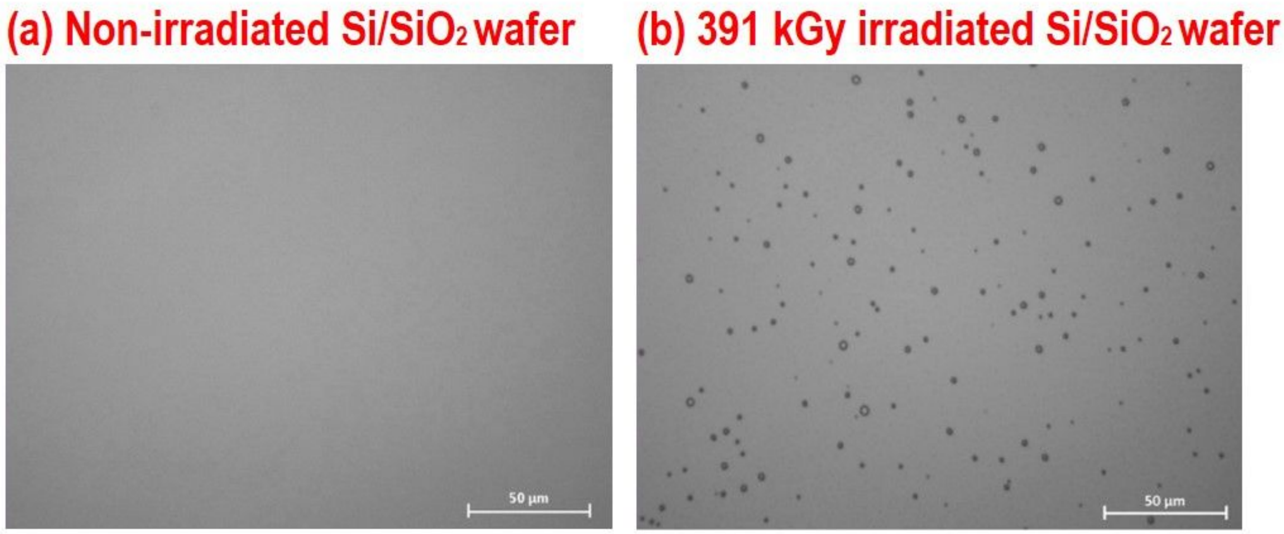

Figure S6: Optical micrographs of a SiO_2/Si wafer: (a) prior to irradiation and (b) after irradiation to an absorbed dose of 391 kGy. All scale bars correspond to a length of 50 µm.

5. Correlative Optical Microscopy and Raman/PL Mapping of Few-Layer MoS_2 Crystals

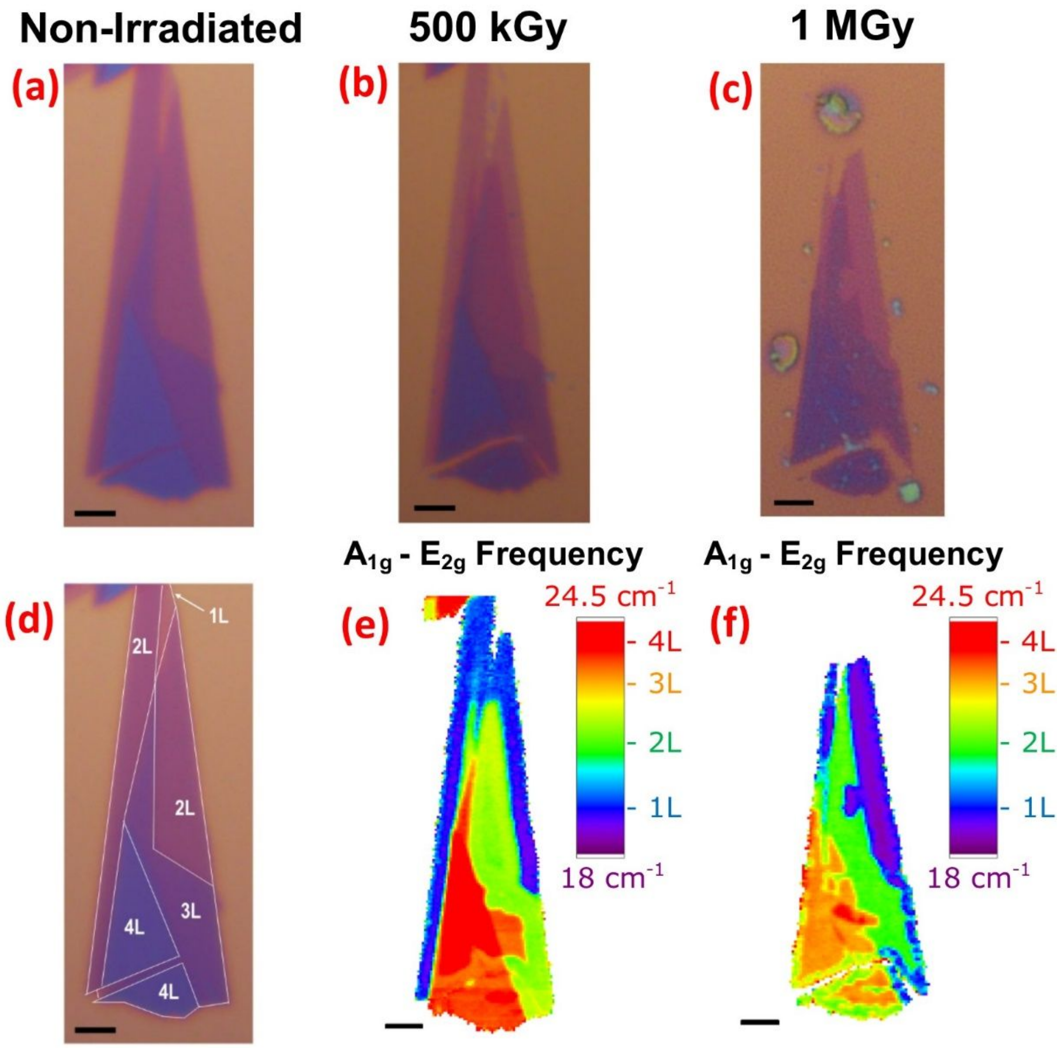

Figure S7: Optical micrographs of MoS_2 crystals, produced by micromechanical exfoliation, **(a)** prior to irradiation and after irradiation to absorbed doses of **(b)** 500 kGy and **(c)** 1 MGy. **(d)** Annotated optical micrograph of the MoS_2 crystals prior to irradiation showing the mono- (1L), bi- (2L), tri- (3L) and quadri-layer (4L) regions. **(e & f)** Correlative Raman maps of the MoS_2 crystals visible in the optical micrographs showing the variation of the $A_{1g} - E_{2g}^1$ frequency difference across the crystals after irradiation to an absorbed dose of **(e)** 500 kGy and **(f)** 1 MGy. Reference labels denoting the typical $A_{1g} - E_{2g}^1$ frequency differences of 1L, 2L, 3L and 4L crystals have been added to the z-axis scale. All scale bars correspond to a length of 2 μm.

Optical micrographs of a two-dimensional (2D) MoS_2 crystal exhibiting 1L, 2L, tri- (3L) and quadri-layer (4L) domains were acquired prior to irradiation (**Figure S7(a)**) and after irradiation to absorbed doses of 500 kGy (**Figure S7(b)**) and 1 MGy (**Figure S7(c)**). Crystal domains of different thicknesses are

identifiable on account of their distinct optical contrast. However, the optical micrograph of the pristine 2D crystal has been annotated to emphasise the contrast of the 1L, 2L, 3L and 4L domains (**Figure S7(d)**). Due to the symmetry of the out-of-plane A_{1g} mode, S atoms in adjacent MoS_2 layers exert an effective restoring force on one another, leading to a redshift of the A_{1g} mode in thinner MoS_2 crystals on account of the lower force constant of the vibration.[18] Unlike the A_{1g} mode, the in-plane E_{2g}^{1} mode does not redshift in thinner pristine crystals due to reduced effective restoring forces. Instead, a blueshift is observed and attributed to an increase in the surface force constant. This is related to a slight charge redistribution caused by the absence of neighbouring MoS_2 layers.[19,20] Hence, the A_{1g} - E_{2g}^{1} frequency difference is a reliable metric commonly used to determine the thickness of 2D MoS_2 crystals that has been rigorously verified both experimentally and theoretically.[18,19]

Therefore, in order to assess whether edge-selective radiolytic etching is exclusive to bilayer (2L) crystals (Figure 3, main text) or applies generally to 2D MoS_2, correlative Raman maps of the 2D MoS_2 crystal, imaged *via* optical microscopy, are acquired. The A_{1g} - E_{2g}^{1} frequency difference is calculated and its variation across the crystal after irradiation to absorbed doses of 500 kGy and 1 MGy is shown in **Figure S7(e)** and **Figure S7(f)**, respectively. Note that, in order to aid interpretation of the Raman maps, 1L, 2L, 3L and 4L reference labels have been added to the A_{1g} - E_{2g}^{1} frequency difference z-axis scale as a guide for the eye.

Pristine 2L MoS_2 exhibits an A_{1g} - E_{2g}^{1} frequency difference of ~ 21 cm^{-1}.[18] However, after irradiation to an absorbed dose of 500 kGy, the frequency difference at the periphery of the 2L domain is just ~ 19 cm^{-1} (Figure S6(e)). This is consistent with edge-selective radiolytic etching of 2L crystals to yield 1L domains and proceeds *via* the mechanism outlined in the main text.

Pristine 3L MoS_2 exhibits an A_{1g} - E_{2g}^{1} frequency difference of ~ 23 cm^{-1}.[18] It can be seen that the 3L region present prior to irradiation is etched to yield 2L MoS_2 after irradiation to an absorbed dose of 500 kGy. This change in morphology is most easily visualised by noting how small the domain size exhibiting

a A_{1g} - E_{2g}^{1} frequency difference of ~ 23 cm^{-1} is after 500 kGy irradiation (Figure S6(e)); relative to the size of the 3L domain prior to irradiation (Figure S6(d)).

Previous correlative atomic force microscopy measurements suggest that domains exhibiting an A_{1g} - E_{2g}^{1} frequency difference of ~ 23.8 cm^{-1} can be assigned as 4L MoS_2.[4] The majority of the 4L regions present prior to irradiation (Figure S6(d)) are etched to yield 3L MoS_2 after irradiation to an absorbed dose of 1 MGy (Figure S6(f)). Moreover, at high absorbed doses, radiolytic etching is not limited to just the uppermost layer of 2D MoS_2 crystals. For example, at the periphery of the 4L and 3L domains, the crystal thickness is observed to reduce by more than one monolayer i.e. etching of 4L→2L and 3L→1L is observed (Figure S6(f)).

For completeness, correlative optical microscopy and Raman/photoluminescence (PL) maps are available for the same crystal and absorbed doses showing the variation of the A_{1g} frequency (**Figure S8**), E_{2g}^{1} frequency (**Figure S9**), PL intensity (**Figure S10**) and PL peak centre (**Figure S11**) across the irradiated 2D MoS_2 crystal. Observations are summarised as follows: (1) after irradiation to an absorbed dose of 500 kGy, the PL intensity is observed to increase within the 1L domains formed *via* edge-selective radiolytic etching of the 2L region (on account of an indirect-direct bandgap transition); (2) a significant reduction in the PL intensity is observed after irradiation to an absorbed dose of 1 MGy (on account of an increase in the number of non-radiative decay pathways caused by the presence of mid-gap states introduced by defects); (3) the blueshift of the PL peak position is attributed to p-type doping caused by charge transfer from occupied conduction band states to adsorbed O_2 molecules.

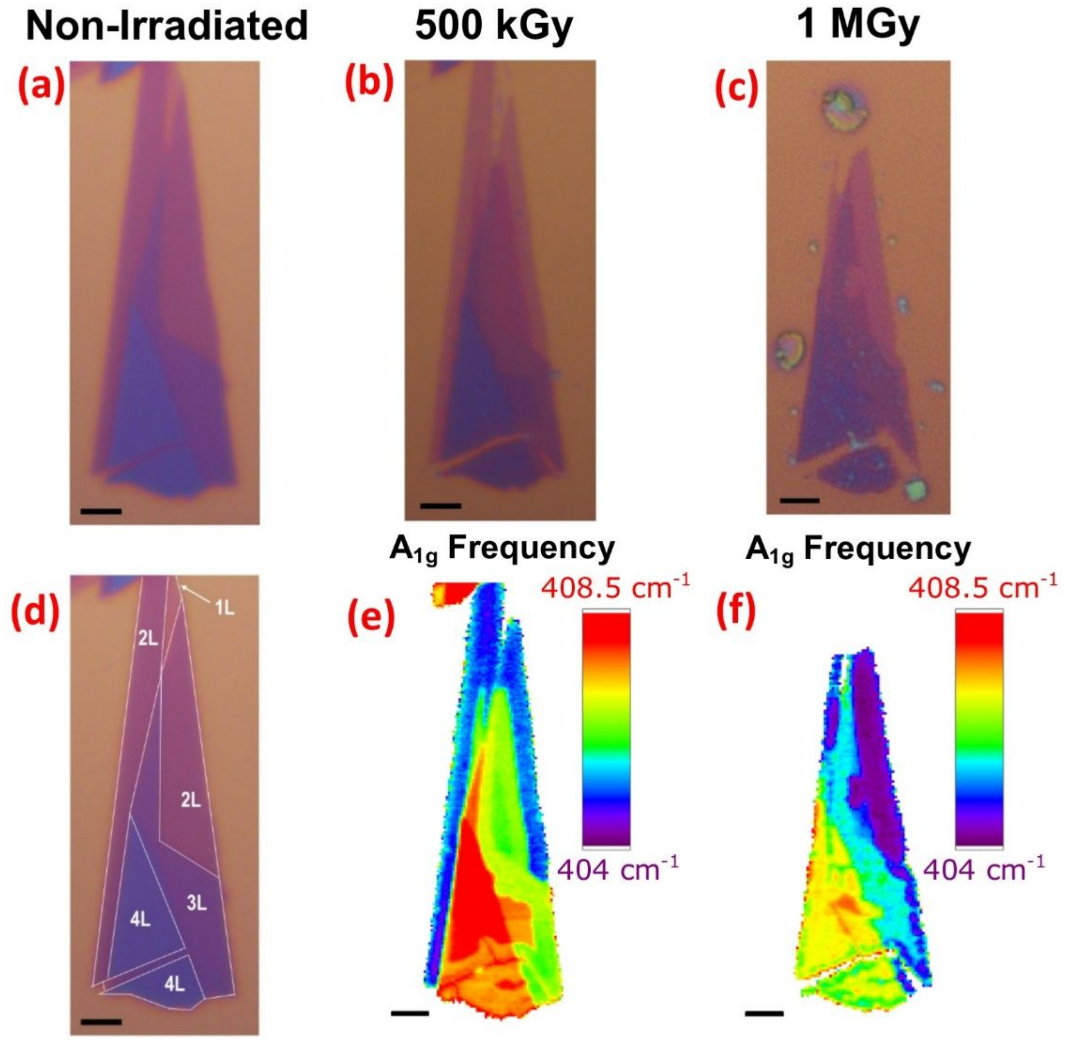

Figure S8: Optical micrographs of MoS_2 crystals, produced by micromechanical exfoliation, **(a)** prior to irradiation and after irradiation to absorbed doses of **(b)** 500 kGy and **(c)** 1 MGy. **(d)** Annotated optical micrograph of the MoS_2 crystals prior to irradiation showing the mono- (1L), bi- (2L), tri- (3L) and quadri-layer (4L) regions. **(e & f)** Correlative Raman maps of the MoS_2 crystals visible in the optical micrographs showing the variation of the A_{1g} frequency across the crystals after irradiation to an absorbed dose of **(e)** 500 kGy and **(f)** 1 MGy. All scale bars correspond to a length of 2 μm.

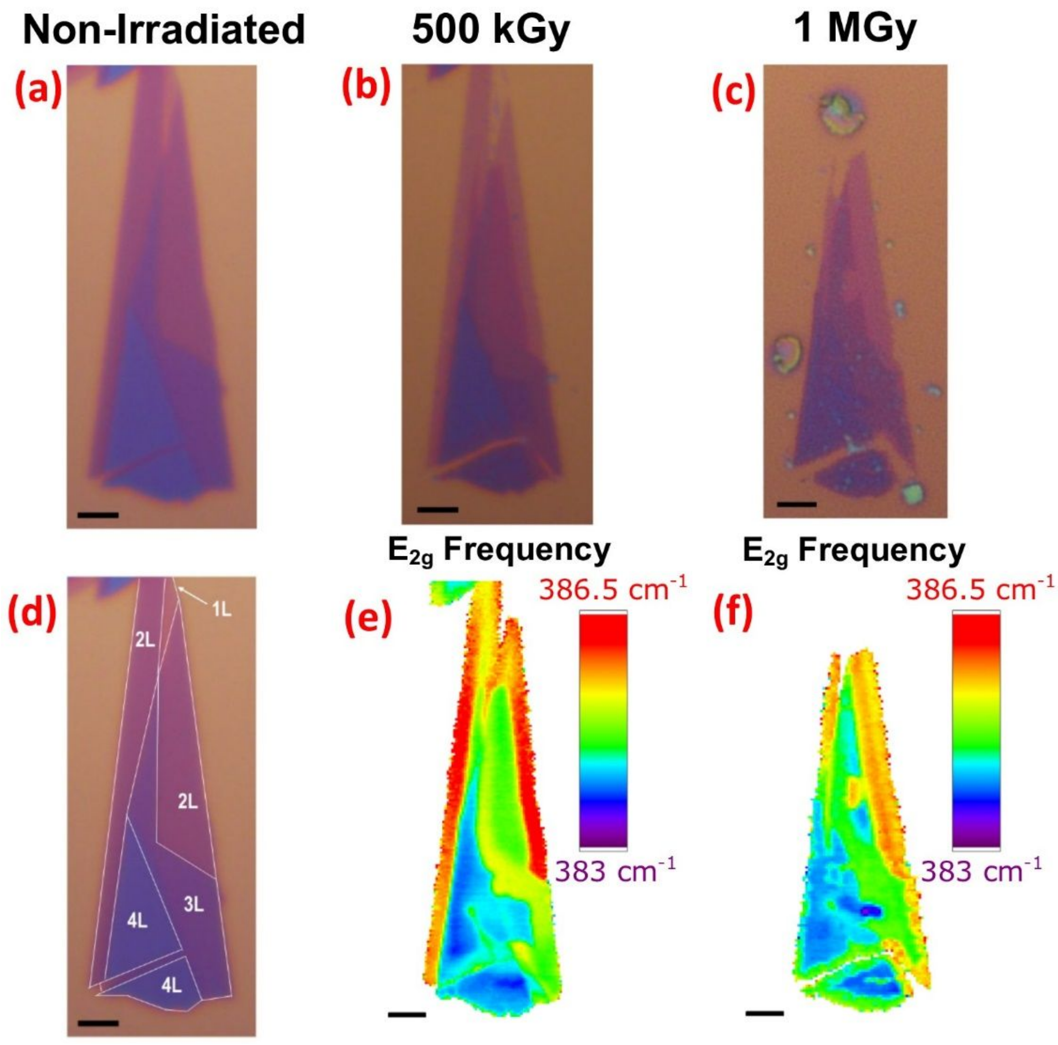

Figure S9: Optical micrographs of MoS_2 crystals, produced by micromechanical exfoliation, **(a)** prior to irradiation and after irradiation to absorbed doses of **(b)** 500 kGy and **(c)** 1 MGy. **(d)** Annotated optical micrograph of the MoS_2 crystals prior to irradiation showing the mono- (1L), bi- (2L), tri- (3L) and quadri-layer (4L) regions. **(e & f)** Correlative Raman maps of the MoS_2 crystals visible in the optical micrographs showing the variation of the E_{2g}^1 frequency across the crystals after irradiation to an absorbed dose of **(e)** 500 kGy and **(f)** 1 MGy. All scale bars correspond to a length of 2 μm.

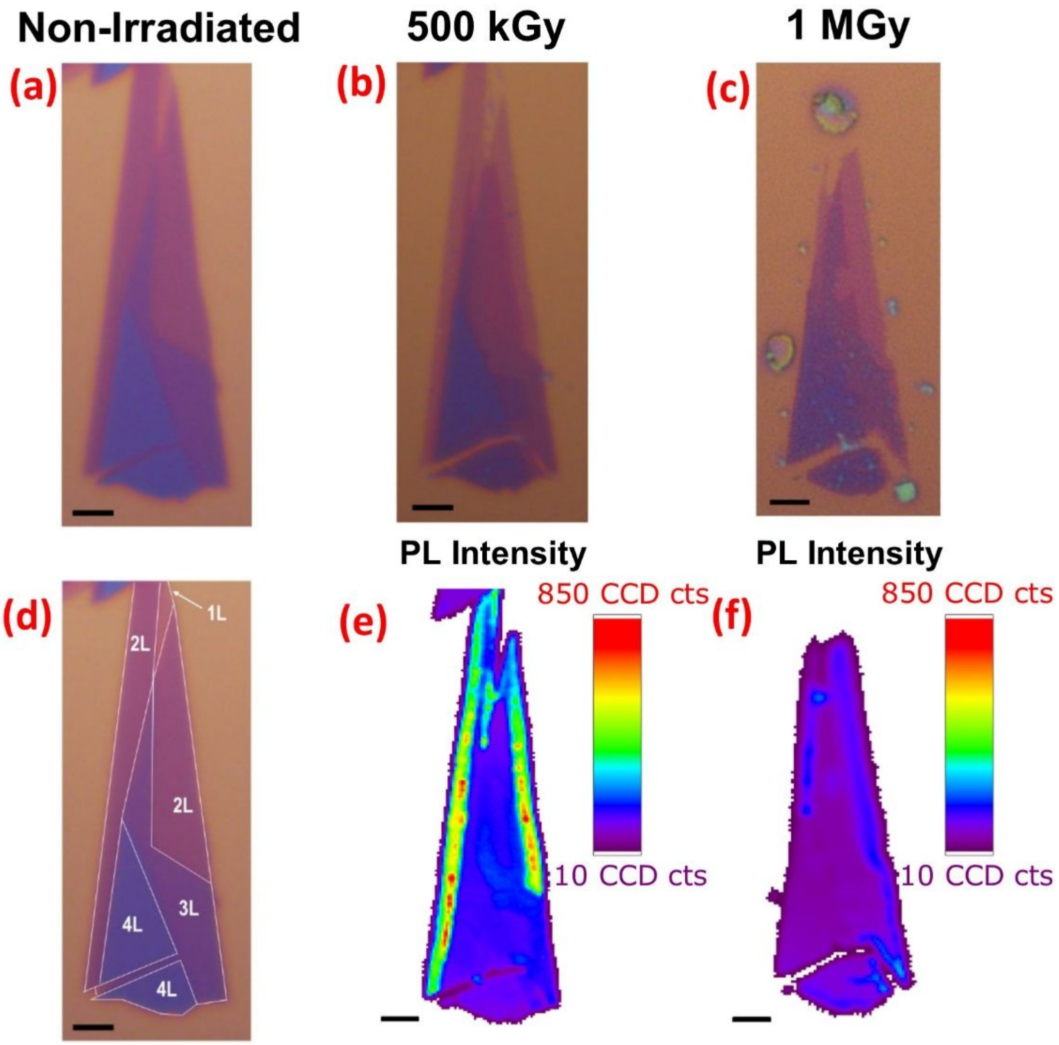

Figure S10: Optical micrographs of MoS_2 crystals, produced by micromechanical exfoliation, **(a)** prior to irradiation and after irradiation to absorbed doses of **(b)** 500 kGy and **(c)** 1 MGy. **(d)** Annotated optical micrograph of the MoS_2 crystals prior to irradiation showing the mono- (1L), bi- (2L), tri- (3L) and quadri-layer (4L) regions. **(e & f)** Correlative photoluminescence (PL) maps of the MoS_2 crystals visible in the optical micrographs showing the variation of the PL intensity across the crystals after irradiation to an absorbed dose of **(e)** 500 kGy and **(f)** 1 MGy. All scale bars correspond to a length of 2 μm.

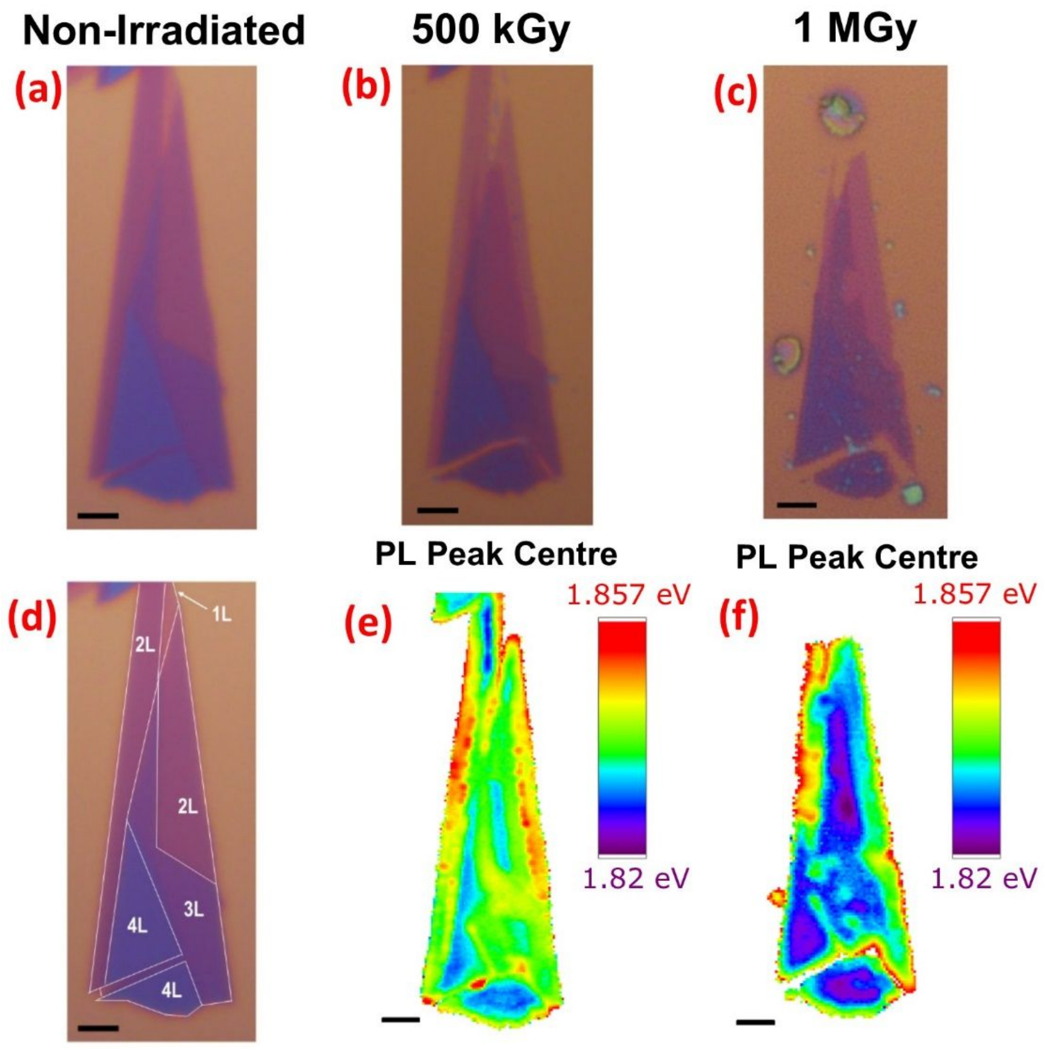

Figure S11: Optical micrographs of MoS_2 crystals, produced by micromechanical exfoliation, **(a)** prior to irradiation and after irradiation to absorbed doses of **(b)** 500 kGy and **(c)** 1 MGy. **(d)** Annotated optical micrograph of the MoS_2 crystals prior to irradiation showing the mono- (1L), bi- (2L), tri- (3L) and quadri-layer (4L) regions. **(e & f)** Correlative photoluminescence (PL) maps of the MoS_2 crystals visible in the optical micrographs showing the variation of the PL peak centre across the crystals after irradiation to an absorbed dose of **(e)** 500 kGy and **(f)** 1 MGy. All scale bars correspond to a length of 2 μm.

6. Full Raman Spectra of 1L MoS_2 Crystals and 1L Border Region after 500 kGy Irradiation

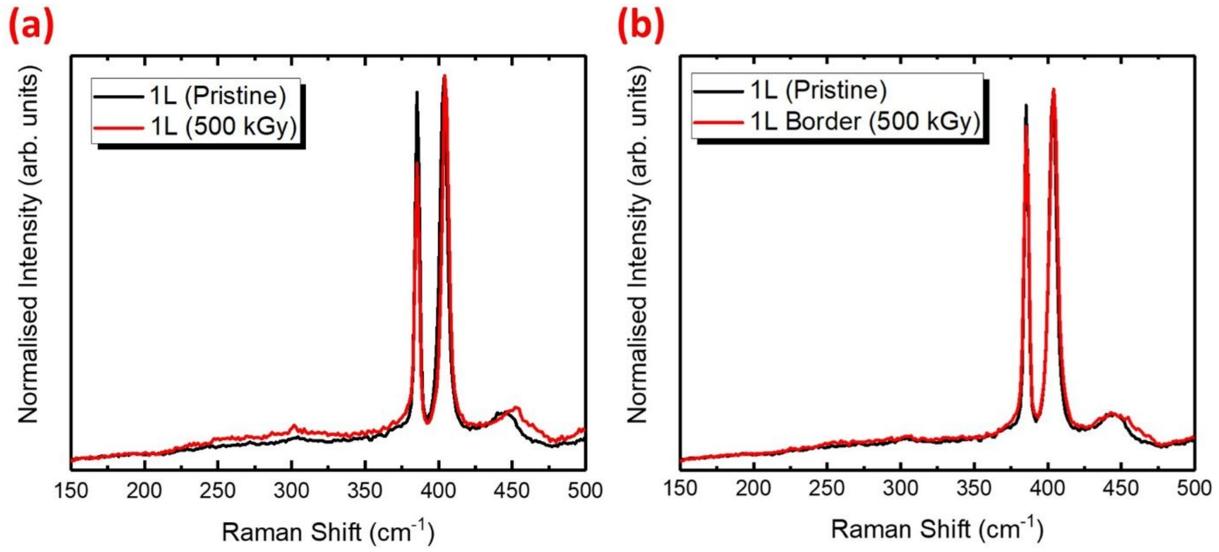

Figure S12: Raman spectra of **(a)** 1L MoS_2 after irradiation to an absorbed dose of 500 kGy and **(b)** 1L MoS_2 produced by edge-selective radiolytic etching of 2L crystals after 500 kGy irradiation, i.e. 1L border. The spectra have been normalised to the intensity of the A_1' signal in pristine 1L MoS_2.

Numerous defect-activated modes are observed in the Raman spectrum of defective 1L MoS_2. The most noteable of these is the $LA(M)$ mode observed at 227 cm^{-1}.[21] **Figure S12(a)** shows that the contribution of this signal towards the Raman spectrum of 1L MoS_2 crystals irradiated to an absorbed dose of 500 kGy is not significant. The scattering process responsible for the $LA(M)$ mode in 1L MoS_2 is described by the phonon-confinement model.[21–23] Consequently, the $LA(M)$ signal becomes observable only in MoS_2 crystals with an average inter-defect spacing of less than 10 nm, i.e. highly defective crystals produced by electron[24] and ion beam irradiation.[25] Therefore, **Figure S12(a)** suggests that the average inter-defect spacing within the basal plane of 1L MoS_2 crystals irradiated to 500 kGy must be greater than 10 nm.

The Raman spectrum of the 1L border region formed after 500 kGy irradiation is provided in **Figure S12(b)**. It is proposed that edge-selective radiolytic etching of the uppermost layer in a 2L crystal yields a border in which the surface of the 1L MoS_2 crystal is covered by oxidised Mo and S species. However, the Raman signals arising from Mo-O bonds[26] are not observed in the Raman spectrum of the 1L border (**Figure S12(b)**). This is attributed to the amorphous morphology and < 1 nm thickness of the oxidised

layer. The fundamental Raman selection rule states that at least one component in the polarizability tensor of a vibrating molecule must change when interacting with the electric field of the incident photon. However, as the polarizability tensor is anisotropic, the cross section for Raman scattering is decreases significantly during a crystalline-to-amorphous transition.[27] Therefore, Mo-O signals are not observed in the Raman spectrum of the 1L border (**Figure S12(b)**) due to the relatively small number and random orientation of Mo-O bonds within the atomically thin amorphous layer. However, the authors prove the existence of Mo-O bonds by using XPS and observing the Mo^{VI} doublet (**Figure 2**, Main Text).

7. Statistical Analysis of Raman/PL Maps of Polycrystalline Monolayer MoS_2 Films

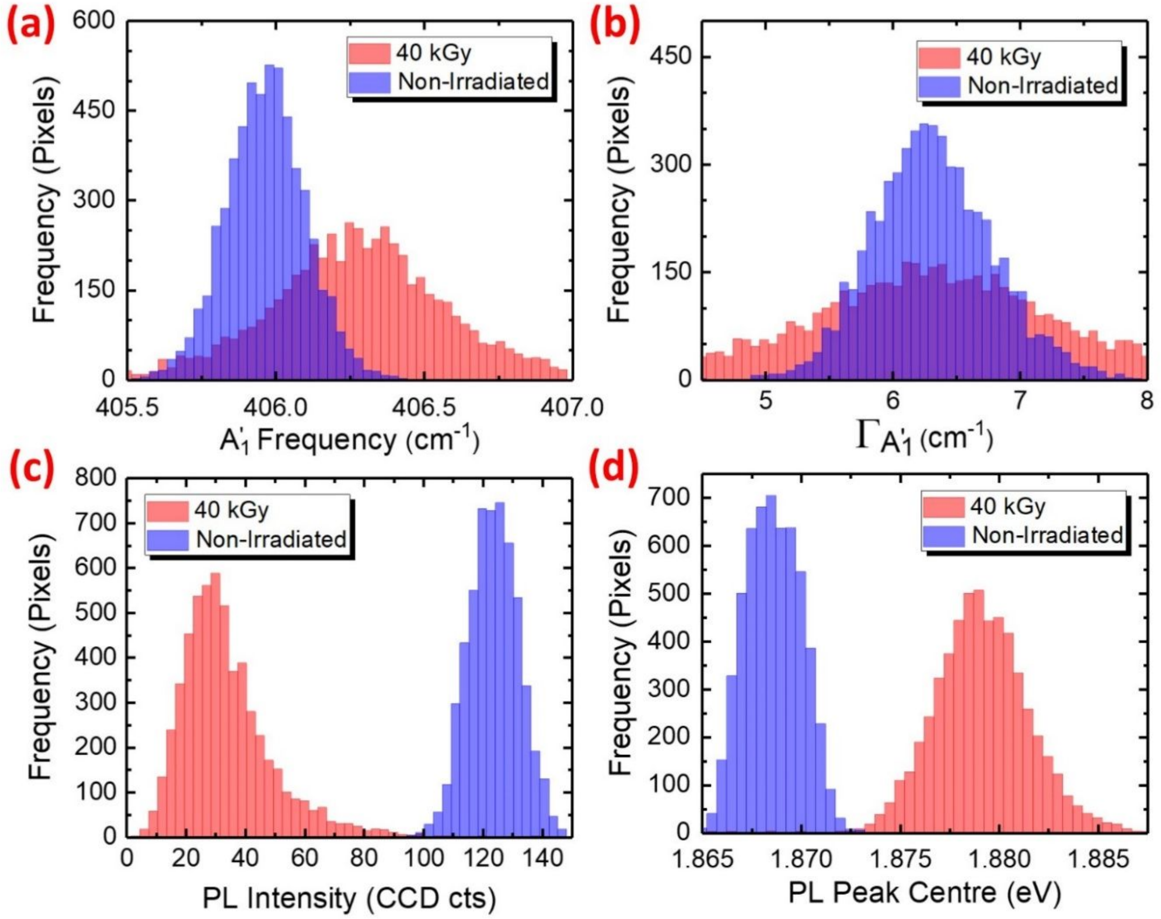

Figure S13: Statistical analysis of Raman and photoluminescence (PL) maps of a polycrystalline monolayer MoS_2 film prior to irradiation and after irradiation to an absorbed dose of 40 kGy. The histograms show the effect of gamma radiation on the **(a)** frequency and **(b)** linewidth, Γ, of the out-of-plane A_1' mode and the **(c)** intensity and **(d)** peak centre of the PL signal.

In order to evaluate the effect of gamma radiation on the vibrational and optical properties of polycrystalline 1L MoS_2 films, Raman and PL mapping is carried out prior to irradiation and after irradiation to an absorbed dose of 40 kGy. Raman and PL maps are acquired over a 15 μm x 15 μm area which constitutes 5625 individual spectra per map i.e. the map is comprised of 5625 pixels. Lorentzian and Gaussian lineshapes are used to fit Raman and PL spectra, respectively.

Figure S13 shows the results of statistical analysis of the Raman/PL maps. The maximum of the out-of-plane A_1' frequency distribution exhibits a slight blueshift upon irradiation to an absorbed dose of 40 kGy (**Figure S13(a)**). However, the resolution of the spectrometer used in this study is ~ 1 cm^{-1} whilst the blueshift of the frequency distribution maximum is < 0.5 cm^{-1}. Moreover, as the kurtosis of the distribution increases upon irradiation, the slight blueshift of the A_1' mode cannot be considered statistically significant. Concerning the linewidth (Γ) of the A_1' signal, the maximum of the distribution does not shift upon irradiation to an absorbed dose of 40 kGy (**Figure S13(b)**). However, a significant increase in the kurtosis of the distribution is observed. This may suggest that the slight blueshift of the A_1' mode could be associated with Γ decreases and increases due to p-doping[25,30] and defect formation,[21] respectively. However, akin to the A_1' frequency distribution, changes in the Γ of the A_1' mode cannot be considered statistically significant on account of the considerable overlap of the pristine and irradiated Γ distributions (**Figure S13(b)**).

Conversely, the optical properties of the polycrystalline 1L MoS_2 film exhibit significant changes upon irradiation to an absorbed dose of 40 kGy. **Figure S13(c)** shows that the PL intensity of the film decreases considerably after exposure to gamma radiation. This is attributed to an increase in the number of non-radiative decay pathways on account of the introduction of defect states within the bandgap of irradiated 1L MoS_2.[25] Moreover, an increase in the energy of the PL signal is observed after gamma irradiation (**Figure S13(d)**). This is attributed to the dissociation of A^- trions into neutral A excitons at higher energy. These p-type changes in the optical properties of the irradiated film are facilitated by charge transfer from occupied conduction band states to adsorbed O_2 molecules, as described in the main text.

However, as the increase in the energy of the PL signal is statistically significant upon irradiation to an absorbed dose of 40 kGy (**Figure S13(d)**), this suggests that the film becomes p-doped and should be accompanied by a significant blueshift and Γ decrease of the A'_1 mode[25,30] which is not observed (**Figures S13(a)** and **S13(b)**). Therefore, the statistically significant shift in the energy of the PL signal could be attributed to the more numerous non-radiative decay pathways of trions.[31] For instance, in pristine materials, the PL intensity of trions is generally lower than excitons. Hence, they do not contribute significantly to the PL signal of 1L MoS_2 films after irradiation due to the overall reduction in PL intensity (**Figure 13(c)**).

8. References

(1) Komsa, H. P.; Kotakoski, J.; Kurasch, S.; Lehtinen, O.; Kaiser, U.; Krasheninnikov, A. V. Two-Dimensional Transition Metal Dichalcogenides under Electron Irradiation: Defect Production and Doping. *Phys. Rev. Lett.* **2012**, 109, 035503.

(2) Benoist, L.; Gonbeau, D.; Pfister-Guillouzo, G.; Schmidt, E.; Meunier, G.; Levasseur, A. X-Ray Photoelectron Spectroscopy Characterization of Amorphous Molybdenum Oxysulfide Thin Films. *Thin Solid Films* **1995**, 258, 110–114.

(3) Madauß, L.; Zegkinoglou, I.; Vázquez Muiños, H.; Choi, Y. W.; Kunze, S.; Zhao, M. Q.; Naylor, C. H.; Ernst, P.; Pollmann, E.; Ochedowski, O.; et al. Highly Active Single-Layer MoS_2 Catalysts Synthesized by Swift Heavy Ion Irradiation. *Nanoscale* **2018**, 10, 22908–22916.

(4) Isherwood, L. H.; Hennighausen, Z.; Son, S.-K.; Spencer, B. F.; Wady, P. T.; Shubeita, S. M.; Kar, S.; Casiraghi, C.; Baidak, A. The Influence of Crystal Thickness and Interlayer Interactions on the Properties of Heavy Ion Irradiated MoS_2. *2D Mater.* **2020**, 7, 035011.

(5) McDonnell, S.; Addou, R.; Buie, C.; Wallace, R. M.; Hinkle, C. L. Defect-Dominated Doping and Contact Resistance in MoS_2. *ACS Nano* **2014**, 8, 2880–2888.

(6) Avval, T. G.; Cushman, C. V.; Bahr, S.; Dietrich, P.; Meyer, M.; Thißen, A.; Linford, M. R. Dimethyl Sulfoxide by Near-Ambient Pressure XPS. *Surf. Sci. Spectra* **2019**, 26, 014020.

(7) Tong, J.; Han, X.; Wang, S.; Jiang, X. Evaluation of Structural Characteristics of Huadian Oil Shale Kerogen Using Direct Techniques (Solid-State ^{13}C NMR, XPS, FT-IR, and XRD). *Energy & Fuels* **2011**, 25, 4006–4013.

(8) Castner, D. G.; Hinds, K.; Grainger, D. W. X-Ray Photoelectron Spectroscopy Sulfur 2p Study of

Organic Thiol and Disulfide Binding Interactions with Gold Surfaces. *Langmuir* **1996**, 12, 5083–5086.

(9) Manocha, A. S.; Park, R. L. Flotation Related ESCA Studies on PbS Surfaces. *Appl. Surf. Sci.* **1977**, 1, 129–141.

(10) DellaGuardia, R. A.; Johnston, F. J. Radiation-Induced Reaction of Sulfur and Water. *Radiat. Res.* **1980**, 84, 259.

(11) Wahlqvist, M.; Shchukarev, A. XPS Spectra and Electronic Structure of Group IA Sulfates. *J. Electron Spectrosc. Relat. Phenom.* **2007**, 156–158, 310–314.

(12) Jaramillo, T. F.; Jorgensen, K. P.; Bonde, J.; Nielsen, J. H.; Horch, S.; Chorkendorff, I. Identification of Active Edge Sites for Electrochemical H_2 Evolution from MoS_2 Nanocatalysts. *Science* **2007**, 317, 100–102.

(13) Xie, J.; Zhang, H.; Li, S.; Wang, R.; Sun, X.; Zhou, M.; Zhou, J.; Lou, X. W. D.; Xie, Y. Defect-Rich MoS_2 Ultrathin Nanosheets with Additional Active Edge Sites for Enhanced Electrocatalytic Hydrogen Evolution. *Adv. Mater.* **2013**, 25, 5807–5813.

(14) He, Y.; Tang, P.; Hu, Z.; He, Q.; Zhu, C.; Wang, L.; Zeng, Q.; Golani, P.; Gao, G.; Fu, W.; et al. Engineering Grain Boundaries at the 2D Limit for the Hydrogen Evolution Reaction. *Nat. Commun.* **2020**, 11, 57.

(15) Fan, X.; Fang, G.; Qin, P.; Sun, N.; Liu, N.; Zheng, Q.; Cheng, F.; Yuan, L.; Zhao, X. Deposition Temperature Effect of RF Magnetron Sputtered Molybdenum Oxide Films on the Power Conversion Efficiency of Bulk-Heterojunction Solar Cells. *J. Phys. D. Appl. Phys.* **2011**, 44, 045101.

(16) Qin, P.; Fang, G.; Ke, W.; Cheng, F.; Zheng, Q.; Wan, J.; Lei, H.; Zhao, X. In Situ Growth of Double-Layer MoO_3/MoS_2 Film from MoS2 for Hole-Transport Layers in Organic Solar Cell. *J. Mater. Chem. A* **2014**, 2, 2742.

(17) Bilgin, I.; Liu, F.; Vargas, A.; Winchester, A.; Man, M. K. L.; Upmanyu, M.; Dani, K. M.; Gupta, G.; Talapatra, S.; Mohite, A. D.; et al. Chemical Vapor Deposition Synthesized Atomically Thin Molybdenum Disulfide with Optoelectronic-Grade Crystalline Quality. *ACS Nano* **2015**, 9, 8822–8832.

(18) Lee, C.; Yan, H.; Brus, L. E.; Heinz, T. F.; Hone, J.; Ryu, S. Anomalous Lattice Vibrations of Single- and Few-Layer MoS_2. *ACS Nano* **2010**, 4, 2695–2700.

(19) Luo, X.; Zhao, Y.; Zhang, J.; Xiong, Q.; Quek, S. Y. Anomalous Frequency Trends in MoS_2 Thin Films Attributed to Surface Effects. *Phys. Rev. B* **2013**, 88, 075320.

(20) Molina-Sánchez, A.; Hummer, K.; Wirtz, L. Vibrational and Optical Properties of MoS_2: From

Monolayer to Bulk. *Surf. Sci. Rep.* **2015**, 70, 554–586.

(21) Mignuzzi, S.; Pollard, A. J.; Bonini, N.; Brennan, B.; Gilmore, I. S.; Pimenta, M. A.; Richards, D.; Roy, D. Effect of Disorder on Raman Scattering of Single-Layer MoS_2. *Phys. Rev. B* **2015**, 91, 195411.

(22) Arora, A. K.; Rajalakshmi, M.; Ravindran, T. R.; Sivasubramanian, V. Raman Spectroscopy of Optical Phonon Confinement in Nanostructured Materials. *J. Raman Spectrosc.* **2007**, 38, 604–617.

(23) Wu, J. Bin; Zhao, H.; Li, Y.; Ohlberg, D.; Shi, W.; Wu, W.; Wang, H.; Tan, P. H. Monolayer Molybdenum Disulfide Nanoribbons with High Optical Anisotropy. *Adv. Opt. Mater.* **2016**, 4, 756–762.

(24) Parkin, W. M.; Balan, A.; Liang, L.; Das, P. M.; Lamparski, M.; Naylor, C. H.; Rodríguez-Manzo, J. A.; Johnson, A. T. C.; Meunier, V.; Drndić, M. Raman Shifts in Electron-Irradiated Monolayer MoS_2. *ACS Nano* **2016**, 10, 4134–4142.

(25) He, Z.; Zhao, R.; Chen, X.; Chen, H.; Zhu, Y.; Su, H.; Huang, S.; Xue, J.; Dai, J.; Cheng, S.; et al. Defect Engineering in Single-Layer MoS_2 Using Heavy Ion Irradiation. *ACS Appl. Mater. Interfaces* **2018**, 10, 42524–42533.

(26) Seguin, L.; Figlarz, M.; Cavagnat, R.; Lassègues, J.-C. Infrared and Raman Spectra of MoO_3 Molybdenum Trioxides and $MoO_3{\cdot}xH_2O$ Molybdenum Trioxide Hydrates. *Spectrochim. Acta Part A Mol. Biomol. Spectrosc.* **1995**, 51, 1323–1344.

(27) Zhigunov, D. M.; Kamaev, G. N.; Kashkarov, P. K.; Volodin, V. A. On Raman Scattering Cross Section Ratio of Crystalline and Microcrystalline to Amorphous Silicon. *Appl. Phys. Lett.* **2018**, 113, 023101.

(28) Ozden, B.; Khanal, M. P.; Park, J.; Uprety, S.; Mirkhani, V.; Yapabandara, K.; Kim, K.; Kuroda, M.; Bozack, M. J.; Choi, W.; et al. Raman and X-Ray Photoelectron Spectroscopy Investigation of the Effect of Gamma-Ray Irradiation on MoS_2. *Micro Nano Lett.* **2017**, 12, 271–274.

(29) Greczynski, G.; Hultman, L. X-Ray Photoelectron Spectroscopy: Towards Reliable Binding Energy Referencing. *Prog. Mater. Sci.* **2020**, 107, 100591.

(30) Chakraborty, B.; Bera, A.; Muthu, D. V. S.; Bhowmick, S.; Waghmare, U. V.; Sood, A. K. Symmetry-Dependent Phonon Renormalization in Monolayer MoS_2 Transistor. *Phys. Rev. B* **2012**, 85, 161403.

(31) Jha, P. P.; Guyot-Sionnest, P. Trion Decay in Colloidal Quantum Dots. *ACS Nano* **2009**, 3, 1011–1015.
